# Epigenetic silencing of *HTATIP2* in glioblastoma contributes to treatment resistance by enhancing nuclear translocation of the DNA repair protein MPG

**DOI:** 10.1002/1878-0261.13494

**Published:** 2023-08-09

**Authors:** Thi Tham Nguyen, Premnath Rajakannu, Minh Diêu Thanh Pham, Leo Weman, Alexander Jucht, Michelle C. Buri, Kristof Van Dommelen, Monika E. Hegi

**Affiliations:** ^1^ Neuroscience Research Center and Service of Neurosurgery Lausanne University Hospital (CHUV) and University of Lausanne Epalinges Switzerland; ^2^ Lundin Family Brain Tumor Center Lausanne University Hospital (CHUV) and University of Lausanne Switzerland

**Keywords:** DNA damage repair, epigenetic silencing, GBM, nuclear–cytoplasmic translocation, treatment resistance

## Abstract

Glioblastoma, the most malignant brain tumor in adults, exhibits characteristic patterns of epigenetic alterations that await elucidation. The DNA methylome of glioblastoma revealed recurrent epigenetic silencing of *HTATIP2*, which encodes a negative regulator of importin β‐mediated cytoplasmic–nuclear protein translocation. Its deregulation may thus alter the functionality of cancer‐relevant nuclear proteins, such as the base excision repair (BER) enzyme *N*‐methylpurine DNA glycosylase (MPG), which has been associated with treatment resistance in GBM. We found that induction of *HTATIP2* expression in GBM cells leads to a significant shift of predominantly nuclear to cytoplasmic MPG, whereas depletion of endogenous *HTATIP2* results in enhanced nuclear MPG localization. Reduced nuclear MPG localization, prompted by *HTATIP2* expression or by depletion of MPG, yielded less phosphorylated‐H2AX‐positive cells upon treatment with an alkylating agent. This suggested reduced MPG‐mediated formation of apurinic/apyrimidinic sites, leaving behind unrepaired DNA lesions, reflecting a reduced capacity of BER in response to the alkylating agent. Epigenetic silencing of *HTATIP2* may thus increase nuclear localization of MPG, thereby enhancing the capacity of the glioblastoma cells to repair treatment‐related lesions and contributing to treatment resistance.

## Graphical abstract

Aberrant promoter methylation of HIV‐1 TAT‐interactive protein 2 (*HTATIP2*) attenuates the negative regulatory capacity of glioblastoma cells to reduce nuclear translocation of cancer‐relevant proteins such as the base excision repair enzyme *N*‐methylpurine DNA glycosylase (MPG). Subsequent enhanced nuclear localization of MPG increases the DNA repair capacity and contributes to treatment resistance in glioblastoma.

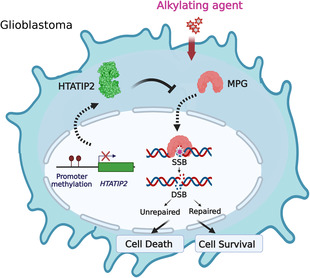

**Abbreviations**
Ab: antibody
AP: abasic site or apurinic/apyrimidinic site
BER: base excision repair
CC3: another name for HTATIP2
DAPI: 4′,6‐diamidino‐2‐phenylindole
DDR: DNA damage response
DMEM: Dulbecco's modified Eagle's mediumDMSO; dimethyl sulfoxide
DNA pol β: DNA polymerase β
Dox: doxycycline
DSB: double‐strand breaks
EGFR: epidermal growth factor receptor
FACS: fluorescence‐activated cell sorting
FBS: fetal bovine serum
GAPDH: glyceraldehyde‐3‐phosphate dehydrogenase
GBM: glioblastoma
GFP: green fluorescent protein
H2AX: H2A histone family member X
H3: histone 3
HRP: horseradish peroxidase
HTATIP2: HIV‐1 Tat‐interactive protein 2
IDEAS: Image Data Exploration and Analysis Software
INI‐43: 3‐(1H‐benzimidazol‐2‐yl)‐1‐(3‐dimethylaminopropyl)pyrrolo[5,4‐b]quinoxalin‐2‐amine
IPTG: isopropyl β‐d‐1‐thiogalactopyranoside
IPZ: importazole
KPNB: karyopherin subunit Β
MGMT: *O*‐6‐methylguanine‐DNA methyltransferase
MMS: methylmethansulfonate
MPG: *N*‐methylpurine DNA glycosylase
MSP: methylation‐specific PCR
NLS: nuclear localization signal
PBS: phosphate‐buffered saline
PCR: polymerase chain reaction
PFA: paraformaldehyde
PI: propidium iodide
PSORTII: protein subcellular localization sites program
qRT‐PCR: quantitative reverse transcription polymerase chain reaction
RT: room temperature
SCE: sister‐chromatid exchange
SDS: sodium dodecyl sulfate
SSB: single‐strand break
TIP30: alternative name for HTATIP2
TLS: translesion DNA synthesis
TMA: tissue microarray
TMZ: temozolomide

## 1 Introduction

Glioblastoma (GBM) is one of the most aggressive malignant brain tumors in adults, notorious for treatment resistance with a median survival of only 15 months. The standard of care relies on genotoxic treatments combining radiotherapy with alkylating agents [1, 2]. No strong pathogenetic dependencies have been identified in GBM, whereas these tumors are characterized by high phenotypic plasticity [3]. Epigenetic deregulation is a key feature of tumor development, enhancing phenotypic plasticity and thereby contributing to all hallmarks of cancer [4]. In an effort to identify epigenetic vulnerabilities in GBM, we identified the HIV‐1 Tat‐interactive protein 2 gene (*HTATIP2*), encoding HTATIP2, also called TIP30 or CC3, as aberrantly methylated. *HTATIP2* promoter methylation has also been reported from other tumor types [5, 6, 7, 8, 9] and found most commonly in GBM and low‐grade glioma, with 70 to over 80% prevalence, according to a pan‐cancer analysis [10]. Loss of HTATIP2 expression has been associated with a broad spectrum of cancer cell type‐dependent biological effects such as loss of pro‐apoptotic properties, enhanced invasion and metastatic potential, and enhanced liver carcinogenesis [11, 12, 13]. In glioma, loss of HTATIP2 expression or epigenetic silencing has been associated with bad prognosis [5, 14].

HTATIP2 has a reported inhibitory function on importin β‐mediated cytoplasmic–nuclear shuttling of cargo proteins [12], suggesting that epigenetic silencing may affect tumor biology by disturbing the balance of nuclear/cytoplasmic localization of a subset of critical proteins. Disturbance of nuclear–cytoplasmic transport has been recognized to be involved in many diseases, including cancer, altering the spatial and temporal location of regulatory proteins [15]. However, little is known about the molecular underpinnings of the various effects attributed to loss of *HTATIP2* expression.

Here, we aimed at elucidating the impact of epigenetic silencing of *HTATIP2* on the biology of glioblastoma, mediated by potential aberrant regulation of subcellular localization of cancer‐relevant proteins. We selected *N*‐methylpurine DNA glycosylase (MPG) as a potentially treatment‐relevant candidate affected by *HTATIP2* silencing in GBM. MPG contains a classic nuclear localization signal (KKQRP), predicting nuclear import via the classic importin α/β1 pathway as suggested by protein localization predictions (WoLF PSORT) [16, 17]. Furthermore, an association of MPG with resistance to alkylating agent therapy in GBM has been reported in a previous study [18]. MPG recognizes DNA damage induced by alkylating agents, *N*‐7‐methylguanine, *N*‐3‐methylguanine, and *N*‐3‐methyladenine that constitute most DNA lesions induced by alkylating agents, and initiates the first step of  base excision repair (BER). Of note, these DNA lesions are not repaired by *O*‐6‐methylguanine‐DNA methyltransferase (MGMT) [19, 20, 21] that plays a pivotal role in resistance to alkylating agent therapy in GBM [22].

Here, we report on a novel mechanism by which *HTATIP2* silencing contributes to treatment resistance of GBM cells to treatment with alkylating agents. Downregulation of HTATIP2 in GBM cells shifted the subcellular localization of MPG from cytoplasmic to nuclear localization, thereby enhancing the DNA repair capacity in response to alkylating agents. This modulation was mediated by the capability of HTATIP2 to block cytoplasmic to nuclear translocation of the DNA repair enzyme MPG.

## 2 Materials and methods

### 2.1 Datasets and data processing

The gene expression data (HG‐133Plus2.0 Affymetrix, Santa Clara, CA, USA) and DNA methylation data H‐450k; Illumina, San Diego, CA, USA) from our GBM patient cohort have been published previously [23, 24] and are available in the Gene Expression Omnibus (GEO) database (http://www.ncbi.nlm.nih.gov/geo/) under the accession numbers GSE7696 and GSE60274. Expression data were normalized by the RMA procedure (r package limma). The CpG probes with detection *P*‐values > 0.01, located on the sex chromosomes, or in SNPs were removed. The functional normalization was performed by the function preprocessFunnorm from the r package minfi [25]. DNA methylation was summarized by beta values [26]. The annotation is based on genome assemblage hg19 (UCSC annotation), and the graphics were produced with r packages gviz, ggplot2, and cowplot. R (http://www.R‐project.org) [27]. Immunohistochemistry and scoring for MPG on a tissue microarray (TMA), of treatment naïve GBM of patients randomized in a clinical trial (EORTC26981/NCIC.CE3) [1], have been described previously [18, 24]. The GBM on the TMA were scored into nuclear (Nuc) MPG (+, weak; ++, strong), cytoplasmic MPG (Cyt), and both nuclear and cytoplasmic MPG (Nuc & Cyt, nuclear with cytoplasmic), or negative for MPG (−) [18], The TMA comprises subsets of samples overlapping with samples mentioned above, for which gene expression and/or methylation data were available from corresponding frozen GBM tissues.

### 2.2 Cell culture

The adherent GBM cell lines LN‐229 (RRID:CVCL_0393), LN‐Z308 (RRID:CVCL_0394), LN‐18 (PRID:CVCL_0392), and LN‐428 (RRID:CVCL_3959) have been established in our laboratory according to institutional directives, approved by the Ethics Committee of the Canton de Vaud (CER‐VD, protocol F25/99) [28]. The GBM cell line BS‐153 (RRID:CVCL_S444) was a kind gift from the laboratory of Adrian Merlo [29]. All cell lines were regularly tested to be mycoplasma‐free (MycoAlert Kit Lonza, Cat. LT07‐418; Lonza, Basel, Switzerland) and were authenticated in 2022 by STR profiling at the Forensic Genetics Unit of the University Center of Legal Medicine, Lausanne, and Geneva [30]. Cells were cultured in Dulbecco's Modified Eagle Medium (DMEM + GlutaMax, 61965‐026; Gibco, Thermo Fisher Scientific, Waltham, MA, USA) supplemented with 5% fetal calf serum (FCS; HyClone, Logan, UT, USA) at 37 °C, 5% CO_2_. Blasticidin (R21001; Thermo Fisher Scientific) and puromycine (P8833; Sigma, Merck, Darmstadt, Germany) 0.5 μg·mL^−1^ were supplemented to maintain selection of transduced cells (inducible cells for HTATIP2, anti‐*MPG*, and anti‐*HTATIP2* shRNAs, respectively). To sustain inducibility and avoid leakiness of the inducible systems over time, we used fresh cells from stock every 2–3 months.

### 2.3 Vector cloning

The recipient vector pCW22 (kindly shared by J. Lingner, EPFL [31]) was digested with Sal‐I and Sbf‐I to remove the *Cas9* gene (4 kb) from the *trAT* (Tet‐On)‐containing plasmid (9.6 kb). The donor vector pEGFP‐C2 that encodes the canonical isoform 1 of HTATIP2 (CC3, UniProtKB/Swiss‐Prot, Q9BUP3‐1) [11] was kindly provided by H. Xiao, University of Michigan. The plasmid was digested by EcoRI and BamHI to isolate the insert, encoding the green fluorescent protein (*GFP*, 717 bp) fused with *HTATIP2* (744 bp), and was gel purified (QIAquick® Gel purification kit; Qiagen, Hilden, Germany). The recipient vector and the isolated *HTATIP2/GFP* sequence were digested with Bam‐II and Sal‐I to generate compatible ends, and the fragments were ligated using T4 DNA ligase (Promega, Madison, WI, USA) (100 mg total DNA/reaction, ratio 1 : 3). Ligation products were transformed into One Shot® TOP10 Chemically Competent *Escherichia coli* (C404010; Thermo Fisher Scientific) using the protocol from the manufacturer. Surviving bacterial colonies were tested with polymerase chain reaction (PCR) for the presence of the *HTATIP2/GFP*‐insert, followed by sequence verification (primer sequences, Table S1). The pCW22 vector containing the *HTATIP2/GFP*‐inducible system was produced in *E. coli* and purified using the QIA Plasmid Miniprep Kit (12163; Qiagen) for lentiviral production.

### 2.4 Lentiviral production and transduction

For lentiviral production, packaging cells, HEK 293 (kindly provided by Tatiana Petrova's Lab), were seeded with 2.5 million cells per 10‐cm petri dish for 24 h (DMEM, 10% FCS). The Lipofectamine 3000 transfection Kit (L3000‐001; Invitrogen, Waltham, MA, USA) was used. In brief, two tubes were prepared, tube A containing 500 μL Optimem medium (31985‐062; Life Technologies, Carlsbad, CA, USA) with 14 μL Lipofectamine 3000, and tube B containing 500 μL Optimem medium, 12 μL P3000 reagent, and three plasmids, with a total of 4.6 ng DNA per petri dish. The three plasmids included the expression vector, the packaging vector—pCMV8.74 (22036; Addgene, Watertown, MA, USA) and the envelop vector—pMD2.G (12259; Addgene), and were used in a ratio 1 : 3 : 4 by DNA weight. The mixture was incubated at room temperature (RT) for 20 min, and then, the content of tube A was transferred to tube B and incubated for 15 min at RT. The medium was aspirated from the HEK 293 cells, and the final mixture was added to the cells. An additional 2 mL of Optimem was added to cover the cells. Cells were incubated for 6 h before changing with a new complete medium. Virus‐containing medium was harvested after 24 h, passed through a 0.22 μm filter (Milan, Scgpt05re: Merck), and complemented with protamine sulfate (10 μg·mL^−1^). This medium was then added to the target cell plates. Target cells were seeded 24 h before transduction, and cells were subjected to antibiotic selection 2 days after transduction.

### 2.5 Doxycycline inducible system for *HTATIP2*

The transduced cells underwent selection with Blasticidin 10 μg·mL^−1^ (R21001; Thermo Fisher Scientific) for 2 weeks. The cell population was then induced with doxycycline (Dox) (D9891‐1G; Sigma‐Aldrich, Merck) for 48 h, and GFP‐positive single cells were sorted by fluorescence‐activated cell sort (FACS) into a 96‐well plate containing DMEM with 20% FBS. These plates were then maintained in cell culture for 3–4 weeks. Fifty microliters fresh medium was added weekly. Of note, GFP‐tagged HTATIP2 will be turned off after 6 days without Dox. Surviving clones were analyzed by Incucyte Zoom 2016A (Version: 3.4; Essen Instruments, Herdfortshire, UK) for characterization of cell growth, inducibility, and titration of the Dox concentrations. Dox concentration of 250 ng·mL^−1^ was selected to induce *HTATIP2* unless otherwise indicated, mRNA, and protein levels were confirmed by quantitative reverse transcription (qRT) PCR and western blot.

### 2.6 IPTG‐inducible system for anti‐*MPG* shRNA and anti‐*HTATIP2* shRNA

The Isopropyl β‐d‐1‐thiogalactopyranoside (IPTG) inducible system uses IPTG (mimics allolactose), to remove a repressor from the lac operon to induce gene expression. Expression vectors pLKO‐puro‐IPTG‐3xLacO, containing nonspecific control shRNA sequence (SHC332‐1EA; Sigma), or one of the three specific sequences for anti‐*MPG* shRNA (Table S2) obtained (Sigma‐Aldrich; Trust in MISSION® Custom Services) and were transfected into TOP10 bacteria to amplify and purify plasmids. Lentiviral production was performed as described above; target cells were LN‐229‐C25^HTATIP2Dox^ and BS‐153‐C01^HTATIP2Dox^ (derived with the procedure described in the previous paragraph). Successfully transduced cells were selected by puromycin (P8833; Sigma) 0.5 μg·mL^−1^. Cells were treated with increasing concentrations of IPTG 0, 250, 500, 1000 μm for 48 h, and MPG expression levels were determined by western blot. The same procedures were followed for the transduction of cells with endogenous HTATIP2 expression (LN‐428, LN‐Z308) using IPTG‐inducible anti‐*HTATIP2* shRNAs (Table S2).

### 2.7 siRNA transfection

Cells with endogenous HTATIP2 expression (e.g., LN‐428 and LNZ‐308) were transfected with anti‐*HTATIP2* siRNA using the Neon electroporation system and kit (MPK‐10025; Thermo Fisher Scientific) according to the manufacturer's recommendations (1400 V, 2 ms, 1 pulse). The siRNAs included Silencer™ Select Negative Control No. 1 siRNA (4390843; Thermo Fisher Scientific), and the specific anti‐HTATIP2 siRNAs: s30128, s30129, and s30700 (4392420; Thermo Fisher Scientific).

### 2.8 Live‐cell imaging

Live‐cell imaging was performed using IncuCyte Zoom S3 2016. Cells were seeded into 96‐well plates (3596; Corning, Merck, Darmstadt, Germany) at a density of 2500 cells/well (LN‐229‐C25) and 3000 cells/well (BS‐153‐C01), respectively. After 24 h, the cells were treated as indicated in the respective experiments. For cell growth and cell death, the cells were subjected to pretreatment with Dox or ITPG for 48 h to induce the *HTATIP2* or the shRNA of interest, followed by methylmethansulfonate (MMS) (129925‐5G; Sigma) treatment. For monitoring of cell death, IncuCyte™ Cytotox Red Reagent (4632; Essen BioScience, Herdfortshire, UK) was added, to the plate at a final concentration of 250 nm immediately after MMS treatment. The plate was then transferred into the IncuCyte incubator, and the cells were monitored by taking images at a 10× magnification every 2 or 3 h for 4 days in different channels. Phase contrast was used to determine cell proliferation, green, for the detection of GFP‐tagged HTATIP2, and the red channel for determination of cell death (Cytotox). Each value is the mean of three technical replicates. The experiments were repeated three times.

### 2.9 Protein extraction and western blot

The cells were trypsinized for 2 min, neutralized with cold medium, centrifuged, and the cell pellet was snap‐frozen in liquid nitrogen, and stored at −80 °C until further use. Western blots were performed as described previously [32]. In brief, protein extracts (20–40 μg) were separated on SDS (sodium dodecyl sulfate) polyacrylamide gradient gels (4–20%, 456‐1086; Bio‐Rad, Cressier, Switzerland) and transferred to a nitrocellulose 0.45 μm blotting membrane (162‐0115; Bio‐Rad). The membranes were incubated with respective primary antibodies (Abs) overnight at 4 °C and subsequently with the corresponding secondary HRP‐conjugated (Horseradish Peroxidase) Abs for 45 min at RT. The list of primary and secondary Abs, and their dilutions are specified in Table S3.

### 2.10 Nuclear and cytoplasmic fractionation

Cells were seeded on 10 cm petri plates at a density of 0.8 million cells/plate. After 24 h, the medium was changed, and cells were induced with 250 ng·mL^−1^ Dox for 48 h. The cells were washed with PBS (Phosphate‐Buffered Saline), followed by adding the lysis buffer of the fractionation Kit, complemented with phosphatase inhibitor and protease inhibitors (Thermo Scientific™ Halt™ Protease Inhibitor Cocktail, Halt™ Phosphatase Inhibitor Cocktail) directly on the plate, and kept immediately on ice. The NE‐PER Nuclear and Cytoplasmic Extraction Kit (78833; Thermo Fischer™) was used, according to the manufacturer's instructions, followed by western blot analysis.

### 2.11 RNA extraction, qRT‐PCR

RNA extraction and qRT‐PCR were performed as reported previously [32] using the primer sequences summarized in Table S1. Expression was normalized to the glyceraldehyde‐3‐phosphate dehydrogenase (*GAPDH*) gene.

### 2.12 Methylation‐specific PCR

The extracted DNA was subjected to bisulfite treatment using the EZ DNA Methylation Kit (Zymo Research, Irvine, CA, USA) followed by methylation‐specific PCR (MSP). During the bisulfite treatment, unmethylated cytosine, but not its methylated counterpart, is converted into uracil. The PCR was performed using published MSP primers for methylated and unmethylated alleles, respectively, and resolved on a 3% agarose gel (Table S1) [33].

### 2.13 Confocal microscopy and high‐content screening imaging

Cells were seeded on different formats, 6‐well plates, 8 well‐slides, 6‐channel μ‐Slide (80606‐IBI; Ibidi, Gräfelfing, Germany), and 96‐well plates (Operetta; Perkin Elmer, Waltham, MA, USA) at defined densities. Cells were treated with 250 ng·mL^−1^ Dox, Importin β inhibitors such as INI‐43 (3‐(1H‐benzimidazol‐2‐yl)‐1‐(3‐dimethylaminopropyl)pyrrolo[5,4‐b]quinoxalin‐2‐amine; Sigma, SML1911‐5MG), or Importazole (IPZ, SML0341‐5MG; Sigma) for 48 h. At defined time points, cells were fixed with 4% paraformaldehyde (PFA) (28908; Life Technologies) for 15 min at RT followed by permeabilization with 0.3% Triton X for 15 min at RT, then blocking for 30 min at RT or overnight at 4 °C in blocking buffer (donkey serum 5%, BSA 0.5%, Triton X‐100 0.3%, sodium azide 0.1%, PBS). For staining, plates were incubated overnight at 4 °C with the primary Abs, followed by incubation at RT for 45 min in the dark with the respective secondary Abs (Table S3). Washing with 0.01% Triton X was performed three times after each Ab incubation step to remove excessive Abs. Image acquisition was performed with a Zeiss LSM 880 Airyscan confocal microscope (Zeiss, Munich, Germany) at 40× magnification (Cellular Imaging Facility, UNIL). Settings included four color channels/excitations: DAPI (4′,6‐diamidino‐2‐phenylindole, 408 nm, blue), GFP (488 nm, green), MPG/Alexa Fluor 555 (548 nm, red), P‐H2AX/Alexa Fluor 647, or KPNB1/Alexa Fluor 647 (633 nm, far red). Fifteen images were acquired for each condition for quantification analysis with cell profiler software (Broad Institute of MIT and Harvard, Cambridge, MA, USA).

P‐H2AX signal in response to the treatment of increasing MMS concentrations (0–500 nm) and increasing *HTATIP2* induction (Dox+, 0–500 ng·mL^−1^) was acquired in a 96‐well plate format by high‐content screening imaging (Operetta; Perkin Elmer) at distinct time points. Nine images were acquired per well, one well per treatment condition. Quantification of the P‐H2AX signal was performed by cell profiler software.

### 2.14 Image analysis and data processing

The images acquired with confocal microscopy or high‐content screening (Operetta; Perkin Elmer) were exported as TIF files for cell profiler—an open‐source image analysis software (version 2.2.1, https://www.github.com/CellProfiler/CellProfiler/releases, Broad Institute of MIT and Harvard, Cambridge, MA, USA). In addition, for Operetta, the background was subtracted from the image using imagej (https://www.imagej.nih.gov/ij/) before the analysis with cell profiler. A pipeline including metadata identity, object recognition, and calculation steps was optimized. Metadata preparation and input images received a unique name and using the same name format so that images were recognized by a generic code. Second, for object recognition, nuclei (primary object), cells (secondary object), and cytoplasm (tertiary object) were identified and recognized by optimizing several parameters. For nuclei recognition, nuclei size (70–100 pixels), threshold (Global), threshold method (Otsu, three‐classes, threshold smoothing scale (1.3488), correction (1), bounds of threshold (0.0–1.0), and clumps objects identify (‘intensity’) were applied. For cell recognition, identification method (propagation method) and regularization (0.05) were applied. The cytoplasm was defined by excluding the nucleus area from the cell area. Third, after calculation, a series of parameters of interest was selected and was then exported into Excel format (for P‐H2Ax analysis) or as properties file (nuclear translocation analysis). From the Excel file, the number of cells and integrated intensity of P‐H2Ax were used for analyses. The properties file was subjected to the cell profiler analyst in which a machine‐learning‐based approach is implemented. A training set was built by randomly selecting cells into positive (cytoplasmic MPG) and negative (nuclear MPG) subclasses. The training was repeated until reaching satisfactory accuracy of more than 95%; then, the program was applied to the whole database. After computation, the parameters of interest were selected and exported into Excel format (for P‐H2Ax analysis) or as properties files (nuclear translocation analysis). From the Excel file, the number of cells and integrated intensity of P‐H2Ax were used for analyses. The properties file was subjected to the cell profiler analyst software. As a result, 2 CSV files were generated. From that, the P enrichment score or ratio of cytoplasmic/nuclear MPG [34, 35] was plotted by Prism for the final graph. Accordingly, a positive (cytoplasmic) enrichment score indicated that most cells present in the tested cell population displayed predominantly cytoplasmic MPG localization.

### 2.15 Alkylating agent treatment

Cells were seeded, and Dox or IPTG was added after 24 h at a final concentration of 250 and 500 ng·mL^−1^, respectively, or as indicated for 48 h. New medium, with or without Dox, IPTG, and MMS, at the concentrations indicated, was added. Incubation times were as indicated, considering cell‐doubling times, for example, LN‐229‐C25, cell‐doubling time of 24 h, and BS‐153‐C01, cell‐doubling time of 39 h.

### 2.16 Flow cytometry

Cell death and cell survival were analyzed using the Annexin V Apoptosis Detection Kit APC (88‐8007‐72; Life Technologies) following the manufacturer's instructions. In brief, cells were harvested following trypsin treatment (TrypLE™ Express, 12604013; Thermo Fisher Scientific), centrifuged, and resuspended in 1 mL binding buffer of the Annexin V Apoptosis Detection Kit APC (88‐8007‐72; Life Technologies). One million cells were transferred into an Eppendorf tube and washed again with binding buffer. Samples were supplemented with 5 μL of Annexin V for every 100 μL of cells and incubated at RT for at least 15 min in the dark. DNA staining dye (propidium iodide, PI) was added to the sample just before analysis. Cells treated with 10% dimethyl sulfoxide (DMSO) served as apoptosis‐positive controls, untreated, and unstained cells served as negative controls, single color controls for Annexin V, and PI only were performed. Samples were analyzed by FACS canto‐1 at the FACS Core Facility of the University of Lausanne (UNIL). For FACS analysis, cells were gated into quartiles according to Annexin V and PI signals: double negative for Annexin V and PI (living cells); Annexin V positive (Early apoptosis), PI positive (necrosis), and double positive for Annexin V and PI (late apoptosis).

For the study of MPG localization, P‐H2AX, and the cell cycle profile, cells were harvested, fixed, permeabilized, and stained with antibodies and DAPI. For the quantification of MPG localization (nuclear/cytoplasmic), the MPG/DAPI similarity score was generated for every single cell by ideas software (Amnis, Seattle, WA, USA). The frequency distributions of these scores under all five experimental conditions were plotted together.

### 2.17 ImageStream‐based flow cytometry and FACS

Cells were seeded into 15 cm petri dishes (TPP93150) at 1 million cells/plate for BS153‐C01 or 0.7 million cells/plate for LN‐229‐C25 and treated as indicated. For FACS analysis of P‐H2AX, cells were washed with PBS and trypsinized (TrypLE™ Express) for 2 min maximum. Cells were analyzed on the ImageStreamX MarkII—a flow cytometry‐based imaging technique. For ImageStream analysis of nuclear translocation, cells were scraped immediately on the plate. Two million cells were fixed with 100 μL cytofix/cytoperm (554714; BD Biosciences, Franklin Lake, NJ, USA) for 15 min at RT, then washed with Permwash buffer (554714; BD), and centrifuged at 750 r.p.m. for 5 min to discard supernatant. Cells were then stained with primary antibody in 100 μL 1× Permwash buffer (volume ratio 1 : 200) for 30 min at RT. Added 1 mL 1× Permwash and centrifuged at 500 ***g*** for 5 min to wash cells, and incubated with 100 μL secondary antibody (volume ratio 1 : 300) for 30 min at RT. After washing with Permwash buffer, cells were resuspended in PBS + 2.5% FBS. DAPI, 50 μL 300 nm, was added for at least 10 min or overnight before running FACS analysis.

### 2.18 Comet assay

Cells were harvested at 0.1 million cells in 1 mL medium for the comet assay (LubioScience STA‐355; LubioBScience GmbH, Zurich, Switzerland) following the manufacturer's instructions. Image acquisition was performed on a Leica LMS880 microscope (Leica, Heerbrugg, Switzerland) at 20× magnification, with the orientation of the comet from head to tail (left side to right side). At least 15 images were taken for each condition. Analysis was performed with imagej and opencomet (cometbio.org) an open access analysis tool. After the run, every image was visually inspected to control for correct recognition of the comets; otherwise, the specific cell (comet) was marked and automatically removed from the final results. To evaluate Single Strand Break (SSB) DNA damage, the tail moment, defined as the distance from the center of the head to the center of the tail, was used for quantification, considering the relative DNA migration and DNA in the comet tail. The DNA damage is proportional to the tail moment. Of note, we used alkaline electrophoresis, which transforms AP sites into SSB; therefore, it detects SSB, AP sites, and other alkali‐labile DNA lesions [36].

### 2.19 Statistical analysis of experiments

The data from experiments were analyzed and presented in graphs using graphpad prism software or r (http://www.R‐project.org). The data are presented as a mean value with error bars showing the standard deviation (SD), unless stated otherwise. If the data were not normally distributed, log transformation was performed prior to statistical testing. For statistical tests, we used the student *t*‐test or Wilcoxon test to compare variables between two groups, as indicated, and two‐way ANOVA to compare variables among multiple groups. Statistical significance was defined according to *P*‐values, indicated by asterisks symbols (*). The significance level is indicated by the number of asterisks (*): (*), *P* < 0.05, (**), *P* < 0.01, (***), *P* < 0.001, and (****), *P* < 0.0001.

## 3 Results

### 3.1 Epigenetic silencing of *HTATIP2* and subcellular localization of MPG in GBM

**Fig. 1:**
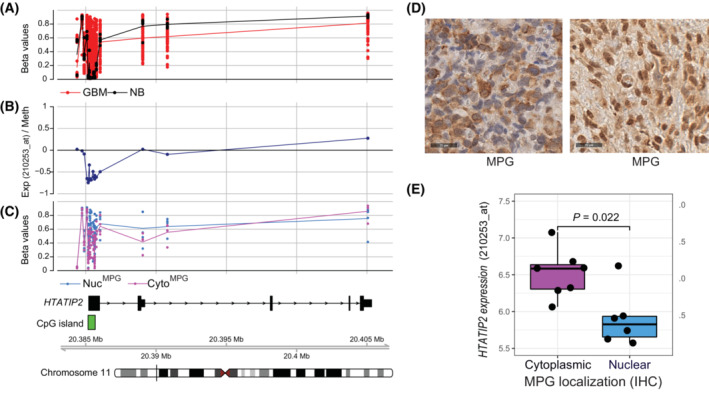
Association of *HTATIP2* methylation and expression with subcellular localization of MPG. (A) The β‐values of CpG methylation in the promoter of *HTATIP2* is visualized for our cohort of GBM (*n* = 63, red) and nontumoral brain (NTB, *n* = 5, black) based on 450k data (chromosomal location of probes as indicated in the track at the bottom: Mb, megabase). Highly variable methylation is observed in the CpG island (indicated in green beneath panel C) associated with the *HTATIP2* promoter in GBM, while no methylation is detected in NTB. (B) The functional methylation of the *HTATIP2* promoter is indicated by a negative correlation (Spearman) between *HTATIP2* expression (Affymetrix probe 210253_at, recognizes all transcripts) and DNA methylation (Exp/Meth) (black). (C) Illustration of CpG methylation of *HTATIP2* (β‐values), stratified by subcellular localization of MPG, nuclear (Nuc, blue), or cytoplasmic (Cyto, pink), of the corresponding tumor tissues as classified by immunohistochemistry (IHC; TMA). (D) MPG expression (IHC, antibody against MPG) in two representative GBM of the cohort displaying either predominantly cytoplasmic (left panel) or nuclear (right panel) MPG, respectively (size bar 25 μm). (E) *HTATIP2* expression (Affymetrix probe 210253_at) was significantly different between GBM samples with cytoplasmic (Cyto, pink) or nuclear MPG (Nuc, blue) (*P* = 0.022, Wilcoxon test), respectively, as determined by IHC of the corresponding samples on the TMA. Cytoplasmic MPG was associated with higher *HTATIP2* expression. Illustrated by boxplot representation, where the rectangle, horizontal dark line, and vertical dark line correspond to the interquartile range (IQR), median, and the distance between minimal and maximal values, respectively. The observations (black points) are superimposed on the boxplot representation. GBM with both, nuclear and cytoplasmic expression were not included in the analyses shown in C and E.

In a genome‐wide DNA methylome analysis of a cohort of GBM, we identified functional methylation of the *HTATIP2* promoter, reflected in a strong negative correlation between expression and methylation (Fig. 1). The reported regulatory function of HTATIP2 on cytoplasmic–nuclear transport of proteins incited us to investigate the repair enzyme MPG as a treatment relevant candidate, based on our previous observation of predominantly nuclear or cytoplasmic localization of MPG in a subset of GBM [18] (Fig. 1). In order to assess a potential association between epigenetic silencing of *HTATIP2* with MPG subcellular localization, we revisited the readings of MPG immunohistochemical staining in our cohort of GBM obtained on a TMA [18, 24]. For the purpose of this analysis, aiming at investigating the functional association of *HTATIP2* silencing and MPG subcellular localization, we focused on GBM that were classified with predominantly nuclear or cytoplasmic MPG expression, respectively (Fig. 1D), excluding samples with both cytoplasmic and nuclear staining (illustrated in Fig. S1), or samples negative for MPG expression. For a limited number of samples, overlapping DNA methylome (HM‐450k) and RNA expression (HG‐133Plus2.0; Affymetrix) data were available. Aberrant methylation of the CpG island, situated in the promoter region of *HTATIP2*, was higher in the GBM classified as expressing nuclear MPG as compared to those with predominantly cytoplasmic MPG expression, as visualized in Fig. 1C. In line, there was a significant difference in *HTATIP2* expression between GBM with nuclear MPG versus cytoplasmic MPG expression (*P* = 0.022, Wilcoxon test). GBM exhibiting nuclear MPG, expressed less *HTATIP2* than those with cytoplasmic MPG (Fig. 1E). Expectedly, GBM with both cytoplasmic and nuclear MPG displayed the full range of *HTATIP2* expression of GBM samples with nuclear or cytoplasmic MPG expression together (Fig. S1).

These findings supported our hypothesis that *HTATIP2* may be involved in regulating the subcellular localization of MPG, thereby influencing the DNA repair capacity of GBM cells and their resistance to alkylating agent therapy.

### 3.2 HTATIP2 expression modulates MPG subcellular localization

**Fig. 2:**
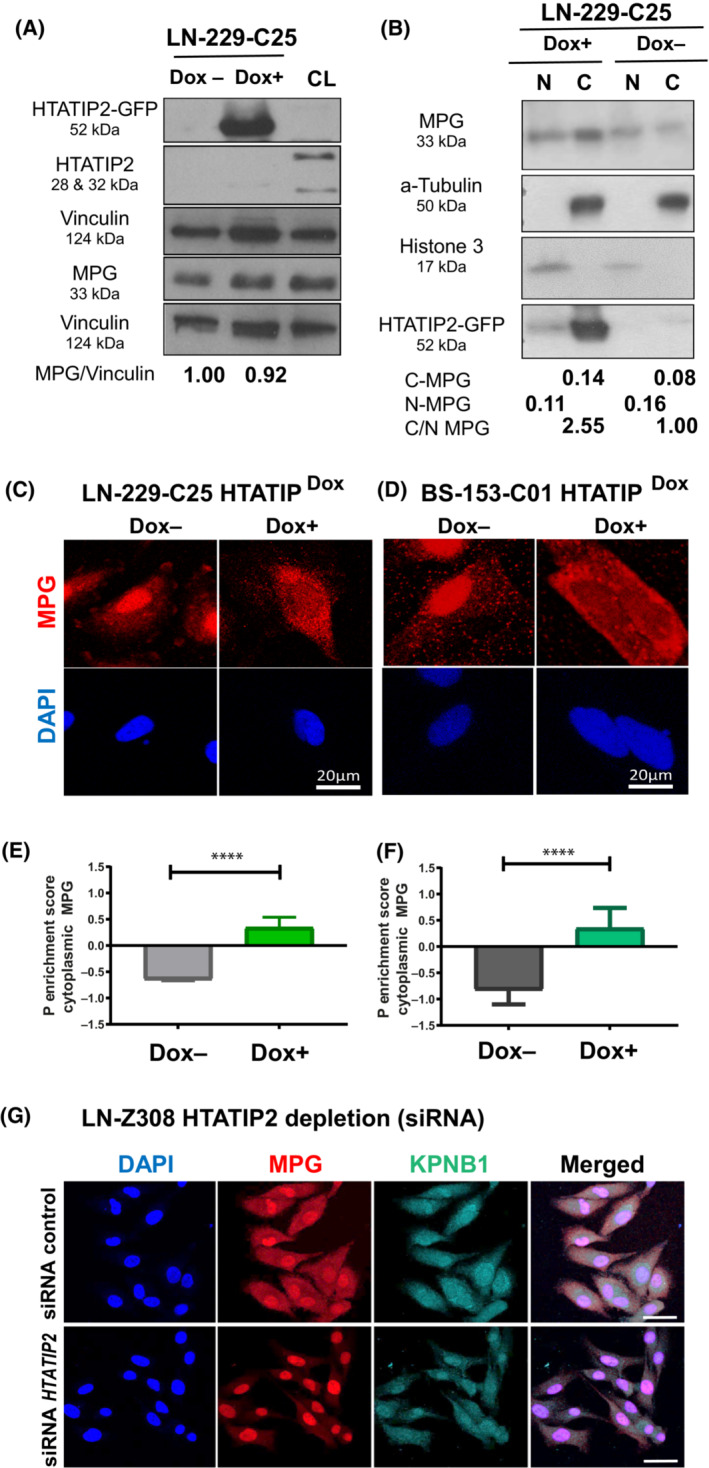
Modulation of HTATIP2 expression affects MPG subcellular localization. (A) Expression of the HTATIP2‐GFP‐fusion protein (52 kDa) was induced by doxycycline (Dox+, 250 ng·mL^−1^) in clone 25 of LN‐229 (LN‐229‐C25‐HTATIP2^Dox^) for 48 h. LN‐229 does not express endogenous HTATIP2. Westerns were probed with antibodies (Abs) against HTATIP2, and MPG (33 kDa), and were normalized to Vinculin (124 kDa) of the corresponding blots. Positive control cell line (CL) for endogenous HTATIP2 (28 and 32 kDa) (LN‐464). MPG expression in presence or absence of HTATIP2‐induction was quantified by densitometry, suggesting no difference in overall MPG expression. (B) LN‐229‐C25‐HTATIP2^Dox^ cells, induced with (Dox+) or not (Dox−), were fractionated and analyzed for relative nuclear (N) and cytoplasmic (C) MPG expression, normalizing the nuclear fraction (N) with Histone 3 (15 kDa) and the cytoplasmic fraction (C) with α‐Tubulin (50 kDa), suggesting increase in cytoplasmic versus nuclear MPG in presence of HTATIP2. The HTATIP2‐GFP‐fusion protein is detected with the anti‐GFP Ab. Representative western blots of three biological replicates are shown in A and B (corresponding full‐length western blots, Fig. S4). (C, D) MPG expression visualized by confocal microscopy (scale bar 20 μm) in cell lines LN‐229‐C25‐HTATIP2^Dox^ and BS‐153‐C1‐HTATIP2^Dox^, respectively, with or without Dox‐induced expression of HTATIP2 (48 h). The corresponding P enrichment scores for cytoplasmic MPG localization were quantified by cell profiler, illustrated in E and F, respectively, for one of three biological replicates (E, *n* = 191 cells; F, *n* = 762 cells; *t*‐test: ****P < 0.0001). Mean values ± SD. (G) The localization of MPG expression is shown for LN‐Z308 upon silencing of endogenous *HTATIP2* after 48 h by siRNA (s700), the most effective of three distinct siRNAs (scale bar 50 μm). Representative images are shown for DAPI, blue; MPG, red; KPNB1, cyan; or merged. LSM 880 microscopy. Cells were visualized by confocal microscopy image, KPNB1‐Alexa 647 (far red), MPG‐Alexa 555 (red), and DAPI (blue). One experiment has been performed, confirming the opposite effect on MPG localization upon knockdown of *HTATIP2* as opposed to induction of *HTATIP2* in LN‐229‐C25, as shown in A–F.

To address the functional relationship between HTATIP2 expression and MPG subcellular localization, we used GBM cell lines (LN‐229, BS‐153) lacking endogenous *HTATIP2* expression due to complete promoter methylation, as determined by MSP (Fig. S2), and transduced them with an inducible, *GFP*‐tagged *HTATIP2* construct (TET‐ON). Both lines displayed a doxycycline (Dox) dose‐dependent induction of HTATIP2‐GFP, while no effect was observed on cell proliferation in the dose range of 0‐500 ng·mL^−1^, monitored by IncuCyte Imaging over a time course of 100 h (Fig. S3). Total MPG was not affected by modulation of HTATIP2 (Fig. 2A). Dox‐induced *HTATIP2* expression in LN‐229‐C25‐HTATIP2^Dox^ led to retention of MPG in the cytoplasm, with reduction of nuclear localization, as demonstrated by nuclear/cytoplasmic fractionation, visualized by western blot and confocal microscopy (Fig. 2B,C) (full‐length western blots corresponding to Fig. 2A,B are shown in Fig. S4). The subcellular localization of MPG in function of HTATIP2‐expression is visualized in an animated 3D projection reconstructed from confocal microscopy (Videos S1 and S2, 3D; DAPI, blue; MPG, red; HTATIP2‐GFP, green). Quantification of the confocal images revealed a significant positive cytoplasmic MPG enrichment score, reflecting MPG retention in the cytoplasm (*P* < 0.0001, paired *t*‐test, Fig. 2E). This was further supported by FACS‐associated imaging (ImageStream), demonstrating a negative shift of the nuclear (DAPI) similarity of MPG localization in presence of Dox‐induced HTATIP2 expression (GFP) (Fig. S5). These results were confirmed in another inducible clone (LN‐229‐C07‐HTATIP2^Dox^, not shown). Similar results were obtained in the GBM cell line BS‐153‐HTATIP2^Dox^‐C01 upon Dox‐induced expression of *HTATIP2* (*P* < 0.0001, unpaired *t*‐test; Fig. 2D,F).

To support the specificity of the observed effect, we performed the reverse experiments, using cell lines expressing endogenous HTATIP2 (LN‐Z308 and LN‐428) and depleting *HTATIP2* with siRNAs^*HTATIP2*^. A shift from predominantly cytoplasmic to nuclear localization MPG was observed in LN‐Z308 upon silencing of endogenous *HTATIP2*, as visualized by immunofluorescence (Fig. 2G). These results were reproduced in LN‐428 (Fig. S6; using three distinct siRNAs and a siRNA‐control, efficiency of up to 90% reduction of *HTATIP2* RNA).

### 3.3 HTATIP2 acts as inhibitor of nuclear receptors attenuating nuclear localization of MPG

To address the question on the mechanism by which HTATIP2 modulates the subcellular localization of MPG, we investigated the role of importin β1 (Karyopherin Subunit Beta 1, KPNB1) that has been reported to physically bind to HTATIP2 [12]. This interaction is predicted to introduce steric hindrance of the cargo binding site of importin β [6].

**Fig. 3:**
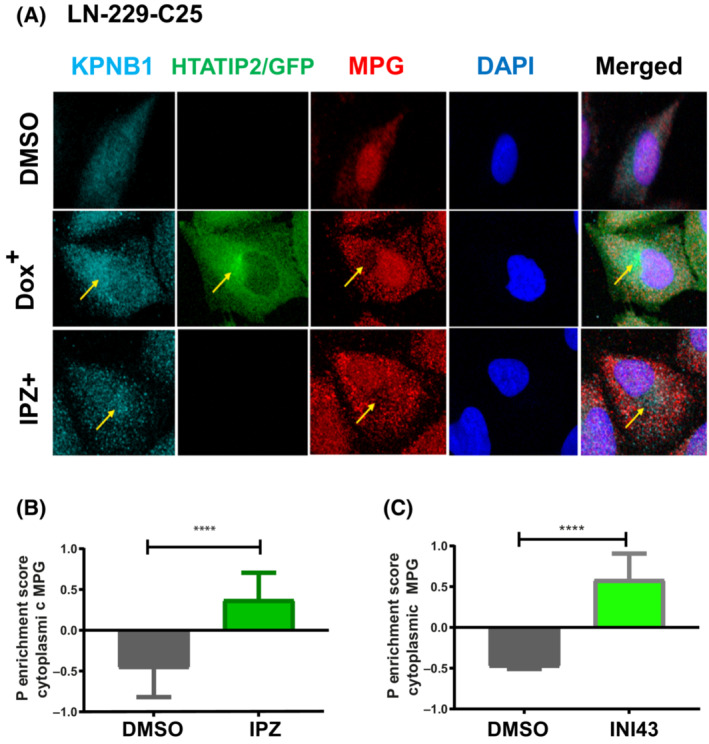
HTATIP2 depletion favors nuclear localization of MPG. (A) LN‐229‐C25‐HTATIP2^Dox^ cells were either induced with Dox to express HTATIP2 or treated with 8 nm importazole (IPZ) for 48 h. Cells were visualized by confocal microscopy image using four channels: KPNB1‐Alexa 647 (far red), GFP (488, green), MPG‐Alexa 555 (red), and DAPI (blue). HTATIP2‐GFP localizes mainly in the cytoplasm, with a focus in proximity to the nuclear membrane. HTATIP2 co‐localized with importin β1 (KBNB1) at the location where MPG was excluded. Areas of interest are indicated by arrows. (B, C) Quantification of the P cytoplasmic enrichment score for MPG upon treatment with the pharmacologic inhibitors of KPNB1: (B) IPZ (8 nm); (C) INI‐43 (8 nm). INI‐43 treatment: *n* = 486 cells, *t*‐test: ****P < 0.0001. IPZ treatment: *n* = 263 cells, *t*‐test: ****P < 0.0001. Representative read‐out of one of three biological replicates, mean ± SD.

HTATIP2 localizes prominently in proximity to the nuclear membrane area and appears to co‐localize with importin β1 (karyopherin subunit beta 1, KPNB1), while excluding MPG from the co‐localization area, as visualized by confocal microscopy (Fig. 3A). To test the involvement of importin β1, we used the pharmacologic inhibitors importazole and INI‐43 that have been shown to interfere with nuclear translocation of importin β1 and thereby block nuclear entry of known cargo proteins of importin β1 [37, 38]. Upon treatment of the cells with either importazole or INI‐43, both, importin β1 and MPG were retained in the cytoplasm, again displaying exclusive localization (Fig. 3A). Hence, in absence of HTATIP2 pharmacologic inhibition of importin β1 induced cytoplasmic retention and exclusive localization of Importin β1 and MPG. The quantification of the subcellular localization of MPG revealed a significant cytoplasmic MPG enrichment score after treatment with the importin β1 inhibitors importazole and INI‐43, hence phenocopying the effect of HTATIP2 expression (both, *P* < 0.0001, unpaired *t*‐test; Fig. 3B,C). Mechanistically, this is consistent with binding of HTATIP2 to importin β1, as has been suggested based on the crystal structure and pull‐down experiments [6, 12] that may cause steric hindrance of the cargo protein binding site of importin β1. Hence, HTATIP2 may compete with MPG for importin β1 binding, thereby attenuating MPG nuclear transport. In accordance, we observed exclusion of MPG from HTATIP2‐importin β1 co‐localization areas. This is in line with previous studies evoking an inhibitory role of HTATIP2 on nuclear translocation of KPNB1 [12], and confirmed by our finding of a significant effect on the cytoplasmic/nuclear ratio of KPNB1 upon induction of HTATIP2 expression (*P* < 0.0001, *t*‐test; Fig. S7). Consistent with this finding, the reverse experiments, knocking down endogenous HTATIP2 in LN‐Z308 and LN‐428, had the opposite effect on MPG and KPNB1 (Fig. 2G; Fig. S6). Together, these results support the notion that HTATIP2 may block nuclear translocation of MPG by sequestering importin β1.

In order to exclude a generalized HTATIP2‐mediated effect on the BER pathway, we determined the effect HTATIP2 on the relative ratio of MPG over DNA Polymerase β (DNA Pol β). The latter conducts a rate‐limiting step in BER (see scheme in Fig. S8) [39]. Nuclear/cytoplasmic fractionation revealed that the MPG/DNA Pol β ratio decreased in the nucleus, while it increased in the cytoplasm of Dox‐induced HTATIP2‐expressing GBM cells (LN‐229‐C25‐HTATIP2^Dox^ and BS‐153‐C01‐HTATIP2^Dox^; Fig. S8), consistent with distinct effects of HTATIP2 on nuclear translocation of MPG and DNA Pol β (relatively smaller effect on DNA Pol β). Furthermore, this suggested a potential decrease in the DNA repair capacity of the BER pathway due to relative reduced nuclear levels of MPG.

### 3.4 HTATIP2 reduces the formation of AP sites/DNA SSB and DSB upon alkylating agent treatment

Next, we aimed at functionally investigating the effect of HTATIP2 on DNA damage response mediated by BER upon treatment with alkylating agents. We used MMS as a model alkylating agent, as it yields mostly lesions with N‐alkylation (95% of the total alkylations, *N*‐7‐methylguanine, *N*‐3‐methylguanine, and *N*‐3‐methyladenine) that are repaired by BER and not by MGMT. These lesions are recognized and hydrolyzed by MPG, yielding AP sites that are subsequently resolved by BER [19, 20, 40]. TMZ also induces these lesions, but to a lesser extent, but yields more O6‐methylguanine that is repaired by MGMT. Hence, to specifically study the role of BER pathway in resistance to alkylating agents, MMS is a good model compound.

**Fig. 4:**
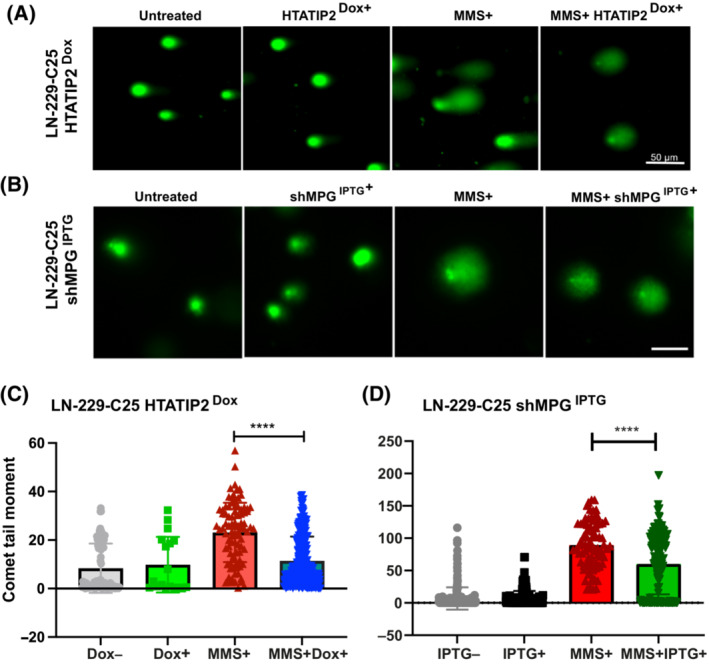
Induction of HTATIP2 or depletion of MPG reduce MMS‐induced SSB and AP sites. The formation of single‐strand breaks (SSBs) and abasic or apurinic/apyrimidinic (AP) sites were evaluated upon induction of HTATIP2 or depletion of MPG using the alkaline comet assay after 48 h of methylmethansulfonate (MMS) treatment. (A) Visualization of the comet tail moment under the four indicated experimental conditions using the Dox‐inducible cell line LN‐229‐C25 for HTATIP2 expression (image scale bar, 50 μm), and (B) the LN‐229‐C25 cell line with IPTG‐inducible anti‐MPG shRNA5^IPTG^ (image scale bar, 50 μm). SSBs and AP sites were quantified as comet tail moment, defined by the distance from the center of the comet head to the center of the comet tail. (C) Analysis was performed on 478 cells (imagej, fiji, adding opencomet analysis tool). Treatment conditions: Untreated, gray; Dox (250 ng·mL^−1^), green; MMS (200 nm), red; and combination, blue. (D) Analysis was performed on a total of 208 images, comprising 713 cells. Treatment conditions: Untreated, gray; IPTG (500 ng·mL^−1^), black; MMS 200 nm, red; and the combination, green (C, D, ****P < 0.0001, *t*‐test, mean ± SD). The experiments were performed in triplicate. Control experiment for anti‐*MPG* shRNA with not‐targeting shRNA, see Fig. S9.

First, we investigated the effect of HTATIP2 on DNA repair activity using the alkaline comet assay that detects SSBs and AP sites. A significant difference (*P* < 0.0001, unpaired *t*‐test) was observed upon MMS treatment for 48 h in absence of HTATIP2 expression, with a longer comet tail moment as compared to HTATIP2‐expressing cells (LN‐229‐C25‐HTATIP2^Dox+^) (Fig. 4A,C). HTATIP2 expression (HTATIP2^Dox+^) on its own had no effect on the tail moment in contrast to MMS alone. To evaluate the involvement of MPG in this process, we depleted MPG using an IPTG‐inducible system for anti‐*MPG* shRNA and evaluated DNA SSB with the alkaline comet assay. The cells were treated with IPTG to induce shRNA5^MPG^ for 48 h, followed by MMS treatment (48 h). The MMS treatment of MPG‐depleted cells revealed a significant difference (*P* < 0.0001, unpaired *t*‐test; Fig. 4B,D), with shorter comet tail moments, suggesting less AP sites/DNA SSB than in presence of MPG. No effect on the comet tail moment was observed upon depletion of MPG in absence of MMS treatment. The induction of the control shRNA^IPTG+^ had no impact on MMS‐induced AP sites/DNA SSB (Fig. S9).

**Fig. 5:**
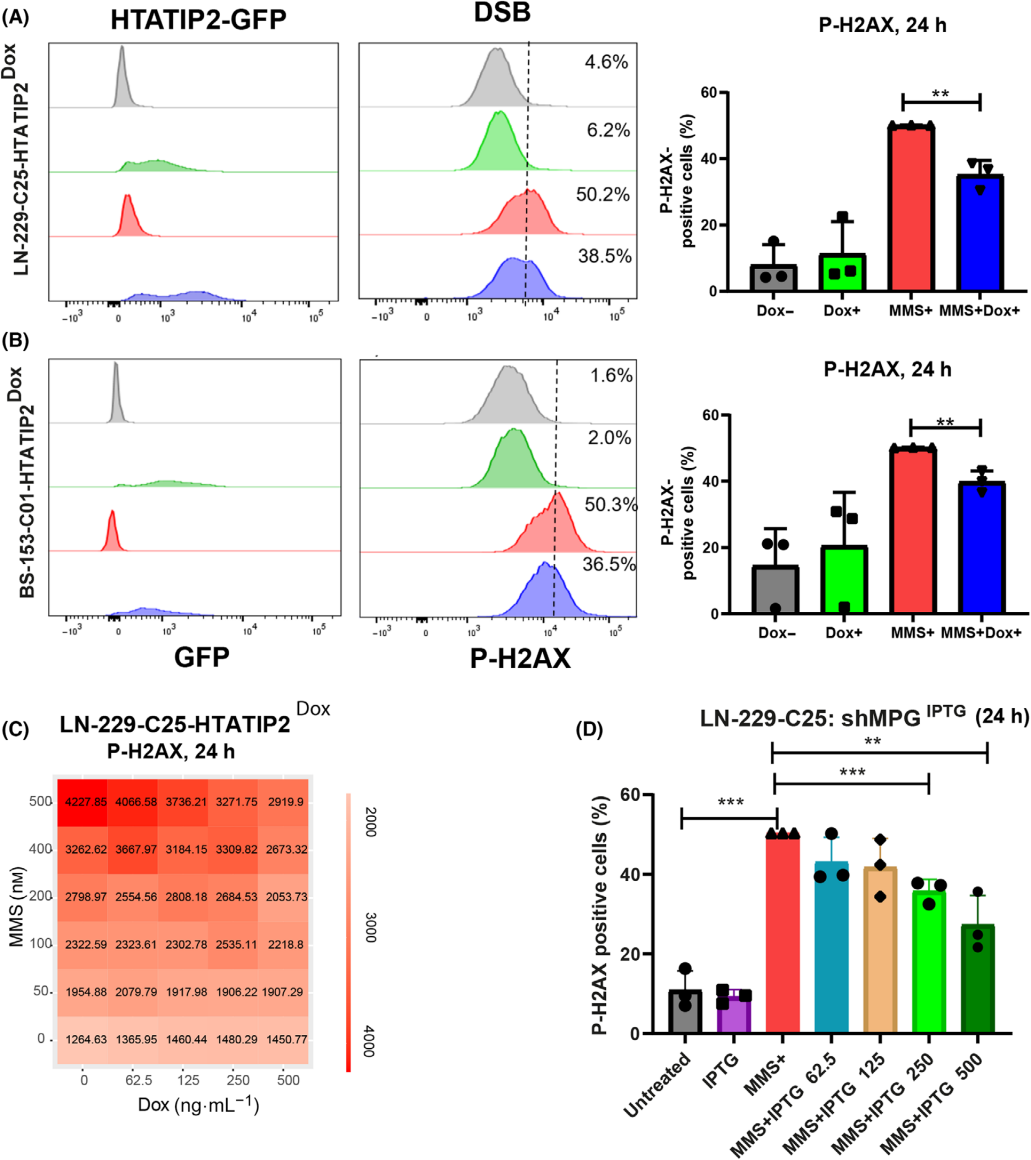
Induction of DSB by MMS in function of HTATIP2 and MPG expression. (A) Expression of HTATIP2‐GFP was induced by Dox in LN‐229‐25‐HTATIP2^Dox^ (250 ng·L^−1^) and (B) BS‐153‐01‐HTATIP2^Dox^ (500 ng·L^−1^) cells for 48 h, followed by methylmethansulfonate (MMS) treatment for 24 h (200 and 100 nm, respectively). Cells were subjected to FACS to quantify the P‐H2Ax signal and monitor HTATIP2‐GFP expression, shown for one representative experiment. The threshold was set at 50% for MMS‐treated cells. The experiments were performed in biological triplicates and quantified as the mean of three biological replicates normalized to MMS+, ±SD (**P < 0.01, *t*‐test). (C) A heatmap of P‐H2Ax signal in LN‐229‐25‐HTATIP2^Dox^ visualizes the response to the combination treatment of increasing concentrations of MMS (0–500 nm) and dose‐dependent induction of *HTATIP2* (Dox 0–500 ng·mL^−1^), quantified 24 h after treatment with MMS (experiment, Operetta high‐content screening). Decreasing levels of DSB were observed with increasing expression of HTATIP2. (D) MPG was depleted in LN‐229‐C25‐shMPG^IPTG^, in absence of HTATIP2, using the inducible shRNA5 against *MPG*. LN‐229‐C25‐sh*MPG* ^IPTG^ cells were pretreated 48 h with ITPG, using a dose range from 0 to 500 ng·mL^−1^, followed by treatment with 200 nm MMS for 24 h. Downregulation of *MPG* significantly altered the formation of DSB, with decreasing P‐H2Ax levels detected by FACS. Depletion of *HTATIP2* significantly affected the formation of MMS‐induced DSB, reflected in increased levels of P‐H2Ax as quantified by FACS. (D) Analysis of three technical replicates (**P < 0.01, ***P < 0.001, unpaired *t*‐test, mean normalized to MMS+, ±SD).

Next, we quantified P‐H2AX, a surrogate marker for DNA damage/DSBs by FACS in LN‐229‐C25‐HTATIP2^Dox^ and BS‐153‐C01‐HTATIP2^Dox^ cells. Treatment of HTATIP2^Dox+^ expressing cells with MMS revealed a difference compared with the un‐induced cells at 24 h as quantified by P‐H2AX (unpaired *t*‐test: *P* = 0.0037, *P* = 0.0056 respectively). A smaller proportion of P‐H2AX positive cells were detected among MMS‐treated, HTATIP2‐expressing cells. No difference was observed in absence of MMS treatment in either cell line (Fig. 5A,B). Again, similar results were obtained upon depletion of *MPG* in LN‐229‐C25 and BS‐153‐C01 with *anti‐MPG* shRNA, followed by MMS treatment. The reduction in MPG resulted in less P‐H2AX positive cells in both cell lines Fig. S10).

In order to evaluate the dose dependence between the expression levels of HTATIP2 and the concentrations of the MMS treatment to induce P‐H2AX expression, HTATIP2 was induced in LN‐229‐C25‐HTATIP2^Dox^ cells with increasing amounts of Dox (0–500 ng·mL^−1^) for 48 h, followed by treatment for 24 h with increasing concentrations of MMS (0–500 nm). Increasing amounts of HTATIP2 resulted in decreasing levels of P‐H2AX in response to the tested dose range of MMS as illustrated in Fig. 5C. Similarly, reduction in MPG in LN‐229‐C25‐shMPG^IPTG^, using increasing concentrations of IPTG (0–500 ng·mL^−1^) followed by MMS treatment, showed less DSB than the corresponding control (Fig. 5D). In accordance, depletion of endogenous *HTATIP2* in LN‐Z308‐shHTATIP2^ITPG^ using an inducible shRNA against *HTATIP2* resulted in an increase in DNA damage upon MMS treatment, as measured by P‐H2AX Fig. S11).

Taken together, these results suggest that HTATIP2 expression retains MPG in the cytoplasm, and thereby decreases nuclear MPG, reducing the DNA repair capacity, reflected in decreased AP sites/SSB or DSB. This effect was phenocopied upon depletion of MPG. In line, the opposite result was observed when knocking down *HTATIP2* in cells with endogenous expression.

### 3.5 MMS effect on proliferation and cell death modulated by HTATIP2 and MPG

**Fig. 6:**
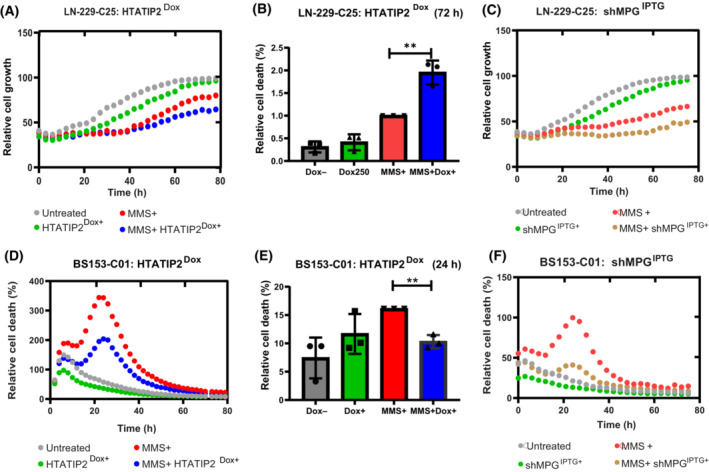
HTATIP2 modulates cell response to MMS treatment. (A) LN‐229‐C25‐HTATIP2^Dox^ cells were treated with Dox for 48 h to induce HTATIP2 expression followed by treatment with methylmethansulfonate (MMS). Relative cell growth was determined by monitoring cell confluency over 72 h by phase contrast, taking images every 3 h (IncuCyte, representative experiment). Treatment conditions, untreated (gray), Dox (250 ng·mL^−1^) (green), MMS (200 nm) (red), and the combination (blue). (B) Cell death at 72 h in response to MMS was detected by Annexin V/APC apoptosis detection kit (**P < 0.01, *t*‐test, based on three biological replicates, mean normalized to MMS+, ±SD). (C) The shMPG was induced in LN‐229‐C25 shMPG^IPTG^ followed by MMS treatment and the relative growth was determined as in (A) (untreated, gray; IPTG [62.5 ng·mL^−1^], green; MMS [200 nm], red; combination, yellow). (D) Relative cell death (Cytotox, red channel IncuCyte, representative experiment) in response to MMS treatment was monitored in BS153‐C01‐HTATIP2^Dox^ over 72 h using the same treatment scheme as in (A) and inducing HTATIP2 with Dox (500 ng·L^−1^). (E) The relative cell death at 24 h was determined with Annexin V/PAC and quantified as in (B) (**P < 0.01, *t*‐test, based on three biological replicates, mean normalized to MMS+, ±SD). (F) Relative cell death in response to MMS was monitored in BS‐153‐C01‐shMPG^IPTG^ as in (D) (Cytotox, red channel IncuCyte) over 72 h upon depletion of MPG (untreated, gray; IPTG [125 ng·mL^−1^], green; MMS [200 nm], red; combination, yellow).

We next studied the effects of HTATIP2 expression on cell proliferation and survival in response to the alkylating agent MMS. We monitored proliferation of LN‐229‐C25‐HTATIP2^Dox^ cells by live‐cell imaging (IncuCyte) and observed decreased cell proliferation upon MMS (200 nm) treatment over the time course of 72 h, with a more pronounced reduction of the growth rate in HTATIP2^Dox+^ expressing cells as compared to their respective controls (Fig. 6A). Follow‐up experiments revealed differences in cell death at 72 h (unpaired *t*‐test, *P* = 0.0033, Fig. 6B), but not at 24 h of MMS treatment, with a higher cell death rate in HTATIP2^Dox+^ expressing cells as compared to HTATIP2 nonexpressing cells, as quantified by FACS Annexin V‐PI analysis. This is in accordance with the separation of the growth curves after only 24 h. In absence of HTATIP2, the depletion of MPG by anti‐MPG shRNA^IPTG+^ phenocopied the effect of HTATIP2^Dox+^ on cell growth in MMS‐treated cells (Fig. 6C). This suggested that the effect of HTATIP2 on cell survival upon MMS treatment is mediated by reduction of nuclear MPG. Taken together, HTATIP2^Dox+^ sensitized LN‐229 to MMS treatment.

### 3.6 HTATIP2 attenuates MMS‐induced cell death at 24 h in BS‐153 cells

The same treatment scheme was applied to BS‐153‐HTATIP2^Dox^. In contrast to LN‐229, we observed an earlier response to MMS treatment, reflected in cell death that peaked at 24 h as monitored by live‐cell imaging, regardless of HTATIP2 expression. The amplitude of cell death was attenuated in HTATIP2‐expressing cells (Dox+) (Fig. 6D), in accordance with the growth curve (Fig. S12). This difference was confirmed by FACS Annexin V‐PI analysis, showing a higher percentage of dead cells after 24 h of MMS treatment in cells not expressing HTATIP2 (Fig. 6E). This pattern of response to MMS treatment was phenocopied by depletion of MPG (shRNA^IPTG+^) in the absence of HTATIP2 expression (Fig. 6F). This suggests that BS‐153 cells were partially protected from toxicity of MMS treatment in presence of HTATIP2 (Fig. 6D,E) or absence/depletion of MPG, respectively (Fig. 6F).

Subsequent cell cycle analyses revealed an expected MMS‐induced disturbance of the cell cycle. We observed a trend (*P* = 0.051) for a HTATIP2‐dependent modulation of the MMS‐induced cell cycle change in LN‐229, with a similar change in the profile upon depletion of MPG. This is compatible with the observed attenuation of MMS associated p21 induction in presence of HTATIP2. For BS‐153, the cycle profiles were not modulated by HTATIP2 expression, or depletion of MPG (Fig. S13). LN‐229 is p53 proficient, in contrast to the other two cell lines.

## 4 Discussion

In this study, we aimed at elucidating the biological role of HTATIP2 in GBM that suffers frequent epigenetic silencing through gene promoter methylation. A regulatory function in cytoplasmic to nuclear translocation of proteins has been attributed to HTATIP2, mediated by physical interaction with importin βs [12]. Therefore, we hypothesized that epigenetic silencing of *HTATIP2* may modulate nuclear transport of cancer‐relevant proteins in an importin β‐dependent manner.

We focused our attention on the DNA repair protein MPG, as we had observed that subsets of GBM presented with almost exclusive nuclear or cytoplasmic MPG localization, respectively. Together with the presence of a classical NLS, predicting nuclear transport via the importin a/β1 pathway, MPG emerged as a promising GBM‐relevant candidate for further investigations. We demonstrated the translocation of MPG from cytoplasmic to predominantly nuclear expression upon *HTATIP2* silencing in several GBM cell lines. The critical role of integrin β1 in this process mediated by HATIP2 was confirmed by the observation that pharmacologic inhibition of importin β1 exerted the same effect as HTATIP2 on cytoplasmic retention of MPG. Hence, providing evidence that epigenetic silencing of *HTATIP2* may represent an underlying mechanism contributing to resistance to alkylating agent therapy that has been associated with nuclear MPG in GBM patients treated in the clinical trial testing TMZ [18]. No such effect was observed in a cohort of GBM patients treated only with radiotherapy before TMZ treatment was introduced as standard of care (pre‐TMZ era) [18]. Supporting mechanistic evidence for the relevance of MPG‐mediated repair in the resistance to the alkylating agents MMS and TMZ has been reported from *in vitro* and respective orthotopic GBM xenograft models using GBM cell lines [18]. Hence, silencing of *HTATIP2* may result in enhanced nuclear MPG, boosting the repair capacity for alkylated DNA lesions, other than *O*‐6‐methylguanine that are rapidly repaired by MGMT, and thereby contributing to treatment resistance.

Conversely, HTATIP2‐induced cytoplasmic retention of MPG decreased the alkylating agent‐induced formation of AP sites and resulting DNA SSBs/DSBs, concordant with reduced P‐H2AX formation in all the models we tested, suggesting attenuated DNA repair capacity. This may attenuate DDR signaling, reducing immediate cell death, and leave behind unrepaired DNA lesions. Subsequent translesion DNA synthesis (TSL)—ignoring DNA lesions—may result in mutations as a trade‐off. Alternatively, unsolved DNA lesions may block the replication fork and may induce sister‐chromatid exchange (SCE)‐mediated repair, resulting in genome instability. However, the consequences on downstream events relevant for cell fate certainly depend in addition on the pathogenetic make‐up of the cells, including, but not limited to p53 function, as reported by others. The HTATIP2‐mediated attenuation of p21 induction by MMS in the p53 proficient cell line LN‐229 may be due to the associated reduction in DNA damage response (DDR) signaling (less P‐H2AX), attenuating the MMS‐induced cell cycle arrest, leaving lesions unrepaired, and subsequently leading to an apparent sensitization of the cells to MMS in presence of HTATIP2. The importance of p53 function for the resistance to alkylating agents has been reported in detail by others, including for LN‐229 [41, 42].

The cellular response to silencing of *HTATIP2* will depend on the cell type‐specific proteome, given its known inhibitory function on a common cellular protein shuttling mechanism (importin a/β1) [12, 15, 43, 44, 45]. Importantly, the effects may only become evident under stress conditions in which HTATIP2‐(de)regulated proteins become rate‐limiting for the stress response. Here, we applied genotoxic stress to the cells by treatment with an alkylating agent that revealed the crucial function of MPG as initiator of BER and its potential role in treatment resistance. Negative regulatory effects of HTATIP2 have been reported for UV‐induced DNA damage repair, affecting respective relevant DNA repair pathways [46]. Other reports implicated HTATIP2 expression in membrane‐cytoplasmic trafficking, resulting in modulation of EGFR signaling [47], and the potential of HTATIP2 overexpression to overcome resistance to the tyrosine kinase inhibitor gefitinib in nonsmall cell lung cancer cells by interfering with EGFR signaling [48].

The magnitude of the HTATIP2‐mediated effect on MPG/BER depends on the extent of promoter methylation and respective reduction in *HTATIP2* expression levels in the cells (Fig. 1C). Clear effects on MPG localization, cytoplasmic or nuclear, were associated with high or low *HTATIP2* expression, respectively. However, immunohistochemical evaluation of MPG in the treatment naïve GBM also identified tumors with both nuclear and cytoplasmic MPG expression patterns that displayed a wide range of *HTATIP2* expression reflecting tumor heterogeneity (Fig. S1). In these cases, the effect of MPG on resistance is certainly less clear and likely contributes to heterogeneous responses of the tumors to alkylating agent therapy. Along the same lines, high and low *HTATIP2* expression that show a statistically significant association with subcellular localization of MPG in GBM (Fig. 1E) will have the strongest impact on treatment response. Unfortunately, due to the lack of power in our gene expression data set of homogenously treated GBM patients, we were not able to establish or disprove an association of *HTATIP2* expression with treatment and outcome.

## 5 Conclusion

Taken together, this study has shed light on a new mechanism contributing to treatment resistance of alkylating agents affecting GBM patients. The epigenetic silencing of *HTATIP2* in GBM abrogates the physiologic negative regulatory effect on nuclear translocation of MPG, enhancing the DNA repair capacity and thereby diminishing the treatment effect. This is a timely finding and may be leveraged by pharmacologic inhibition of importin β1 that phenocopied the effect of HTATIP2 expression in our experiments and may sensitize patients to alkylating agents. Targeting importin β1 has been proposed as treatment for other tumor types, such as prostate cancer and small cell lung cancer, with the aim of attenuating nuclear translocation of cancer‐relevant proteins affecting various biological mechanisms [49, 50]. Targeting nuclear import and export of cancer‐relevant proteins has been recognized as a potential modality for therapy [38, 51]. Preclinical studies evaluate concepts of targeting nuclear import/export to overcome chemotherapy resistance [52, 53]. First results from clinical trials are available from recurrent GBM for an oral exportin‐1 inhibitor [54]. The drug (Selinexor) is currently tested in several trials in combination with other treatments, for example, standard of care (RT/TMZ → TMZ) in the first line and with TMZ in recurrent GBM.

## Conflict of interest

The authors declare no conflict of interest.

## Author contributions

TTN and MEH designed the study. TTN performed the experiments, analyzed and interpreted the data, and wrote the manuscript with MEH. PR and KVD contributed preliminary experimental data. MDTP, LW, AJ, and MCB performed experiments and analyzed data. MEH directed the study. All authors contributed to the manuscript writing.

### Peer review

The peer review history for this article is available at https://www.webofscience.com/api/gateway/wos/peer‐review/10.1002/1878‐0261.13494.

## Supporting information


**Fig. S1.** MPG expression patterns in GBM (related to Fig. 1).
**Fig. S2.** *HTATIP2* promoter methylation in GBM cell lines.
**Fig. S3.** HTATIP2 is induced by Dox in a dose‐dependent manner without affecting cell growth.
**Fig. S4.** Full‐length Westerns corresponding to Fig. 2A and B.
**Fig. S5.** HTATIP2 induces cytoplasmic retention of MPG.
**Fig. S6.** Depletion of *HTATIP2* by siRNA in LN‐428.
**Fig. S7.** HTATIP2 inhibits nuclear transport of KPNB1.
**Fig. S8.** Effect of HTATIP2 on the nuclear and cytoplasmic ratio of MPG/DNA Pol β.
**Fig. S9.** Control anti‐*MPG* shRNA did not affect MMS response in LN‐229‐C25.
**Fig. S10.** Depletion of MPG reduces effect of MMS on DNA DSB.
**Fig. S11.** HTATIP2 depletion enhances the MMS‐induced DNA damage in LN‐Z308.
**Fig. S12.** HTATIP2 attenuates MMS‐induced reduction in cell growth in BS‐153.
**Fig. S13.** Cell cycle progression of MMS‐treated cells in function of HTATIP2.
**Table S1.** List of PCR and methylations‐specific PCR primers.
**Table S2.** Sequences of shRNAs.
**Table S3.** List of antibodies.

**Video S1.** Nuclear localization of MPG in absence of HTATIP2 expression in LN‐229‐C25 (Dox‐) cells (3D).

**Video S2.** Retention of MPG in the cytoplasm upon expression of HTATIP2 in LN229‐C25 (Dox+) cells (3D).

## Data Availability

The datasets used during the current study are available in the Gene Expression Omnibus repository at GEO (http://www.ncbi.nlm.nih.gov/geo/) under the accession numbers GSE7696 and GSE60274.
