# Supplementary material for: Epigenetic silencing of HTATIP2 in glioblastoma contributes to treatment resistance by enhancing nuclear translocation of the DNA repair protein MPG
Source: Mol Oncol. 2023 Aug 9;17(9):1744–62. doi: 10.1002/1878-0261.13494 (PMC10483604; doi:10.1002/1878-0261.13494)
Supplement: Supplementary file 1 — Fig. S1. MPG expression patterns in GBM (related to Fig. 1). Fig. S2. HTATIP2 promoter methylation in GBM cell lines. Fig. S3. HTATIP2 is induced by Dox in a dose‐dependent manner without affecting cell growth. Fig. S4. Full‐length Westerns corresponding to Fig. 2A and B. Fig. S5. HTATIP2 induces cytoplasmic retention of MPG. Fig. S6. Depletion of HTATIP2 by siRNA in LN‐428. Fig. S7. HTATIP2 inhibits nuclear transport of KPNB1. Fig. S8. Effect of HTATIP2 on the nuclear and cytoplasmic ratio of MPG/DNA Pol β. Fig. S9. Control anti‐MPG shRNA did not affect MMS response in LN‐229‐C25. Fig. S10. Depletion of MPG reduces effect of MMS on DNA DSB. Fig. S11. HTATIP2 depletion enhances the MMS‐induced DNA damage in LN‐Z308. Fig. S12. HTATIP2 attenuates MMS‐induced reduction in cell growth in BS‐153. Fig. S13. Cell cycle progression of MMS‐treated cells in function of HTATIP2. Table S1. List of PCR and methylations‐specific PCR primers. Table S2. Sequences of shRNAs. Table S3. List of antibodies. [file MOL2-17-1744-s001.pdf]

Supplementary Tables, Figures and Video legends

Table S1. List of PCR and methylations-specific PCR primers.

Table S2. Sequences of shRNAs.

Table S3. List of antibodies.

Fig. S1. MPG expression patterns in GBM (related to Fig. 1)

Fig. S2. *HTATIP2* promoter methylation in GBM cell lines.

Fig. S3. HTATIP2 is induced by Dox in a dose-dependent manner without affecting cell growth.

Fig. S4. Full length Westerns corresponding to **Fig. 2A** and **B**

Fig. S5. HTATIP2 induces cytoplasmic retention of MPG.

Fig. S6. Depletion of *HTATIP2* by siRNA in LN-428.

Fig. S7. HTATIP2 inhibits nuclear transport of KPNB1.

Fig. S8. Effect of HTATIP2 on the nuclear and cytoplasmic ratio of MPG/ DNA Pol β.

Fig. S9. Control anti-*MPG* shRNA did not affect MMS response in LN-229-C25.

Fig. S10. Depletion of MPG reduces effect of MMS on DNA DSB.

Fig. S11. HTATIP2 depletion enhances the MMS induced DNA damage in LN-Z308.

Fig. S12. HTATIP2 attenuates MMS induced reduction of cell growth in BS-153.

Fig. S13: Cell cycle progression of MMS treated cells in function of HTATIP2.

Video S1. Nuclear localization of MPG in absence of HTATIP2 expression in LN-229-C25 (Dox-) cells (3D).

Video S2. Retention of MPG in the cytoplasm upon expression of HTATIP2 in LN229-C25 (Dox+) cells (3D).

Supplementary Tables

Table S1. List of primers used for PCR and methylation-specific PCR. (MSP)

Gene	Forward primer (5'-3')	Reverse primer (5'-3')
HTATIP2/GFP- insert	AGCGTCGACATGGTGAGCAAG	ATGACCTGCAGGCTAGATCCG
GAPDH	CATGAGAAGTATGACAACAGCCT	AGTCCTTCCACGATACCAAAGT
HTATIP2 (806-807)	AAACCGGCAGAGTGCTCTTA	TTGAAAGGCAGAGGCGTAGT
MPG (1039-1040)	CATTTACGGCATGTACTTCTGC	ATGGTCTCCAGACCTTCCAG
HTATIP2-MSP (methylated [1])	TAGCGTCGTCGCGAGGTTATTCG	CACGACGCAACAAAAACAAAACGAC
HTATIP2-MSP (unmethylated [1])	GTAGTGTTGTTGTGAGGTTATTTGG	CACAACACAACAAAAACAAAACAACC

Table S2. Sequences of shRNAs.

Target Gene	ID/#	**TRC Clone ID	Target Region	Target Sequence
MPG	*MPG-1			ATGTACTTCTGCATGAACATCTCCAGCCA
MPG	*MPG-3			GGCGACTTCCTAATGGCACAGAACTCCGA
MPG	MPG-5	TRCN0000413887	CDS	
HTATIP2	#1	TRCN0000020349	3UTR	
HTATIP2	#2	TRCN0000020353	CDS	
HTATIP2	#3	TRCN0000280399	CDS	

*Sequences from Origene (Cat#TR311419) [2].

**TRC, RNAI CONSORTIUM, target sequences available under TRC clone number at https://www.broadinstitute.org/rnai-consortium/rnai-consortium-shrna-library

TRC clones, and other sh-sequences were custom cloned into the pLKO_IPTG_3xLacO vector, Sigma-Aldrich, Trust in MISSION® Custom Services.

Table S3. List of antibodies

Ab	Code	Company	Species	clonal	kDa	Dilution Western	Dilution IF / IHC
APNG (MPG)	SC-101237	Santa Cruz	Mouse	mAb	33	1:1000	1:200<br>1:100
TIP30 (HTATIP2)	ab125726	Abcam	Rabbit	pAb	28 & 32	1:1000	
DNA pol	ab175197	Abcam	Rabbit	mAb	38	1:1000	
PDI	3501	Cell signaling	Rabbit	pAb			1:100
PARP	9542	Cell signaling	Rabbit	pAb	89,116	1:1000	
P-H2AX	2577L	Cell signaling	Rabbit	pAb	15	1:1000	1:100
KPNB1<br>Importin β1	ab45938	Abcam	Rabbit	pAb	97		1:200
Vinculin	ab129002	Abcam	Rabbit	mAb	124	1:5000	
α-Tubulin	3873S	Cell signaling	Mouse	mAb	50	1:4000	
α-Tubulin	T5168	Sigma	Mouse	mAB	50	1:10000	
GAPDH	G9545	Sigma	Rabbit	pAb	36	1:5000	
Histone 3	ab1791	Abcam	Rabbit	pAb	15	1:5000	
GFP	2956T	Cell signaling	Rabbit	mAb	27	1:2000	
Anti-rabbit<br>HRP Conj	W4011	Promega	Goat	pAB		1:5000	
Anti-mouse<br>HRP Conj	31430	Thermo Fisher	Goat	pAB		1:5000	
Alexa Fluor 555 Anti-mouse	A31570	Thermo Fisher	Donkey				1:300
Alexa Fluor 647 Anti-rabbit	A31573	Thermo Fisher	Donkey				1:300

Supplementary Figures

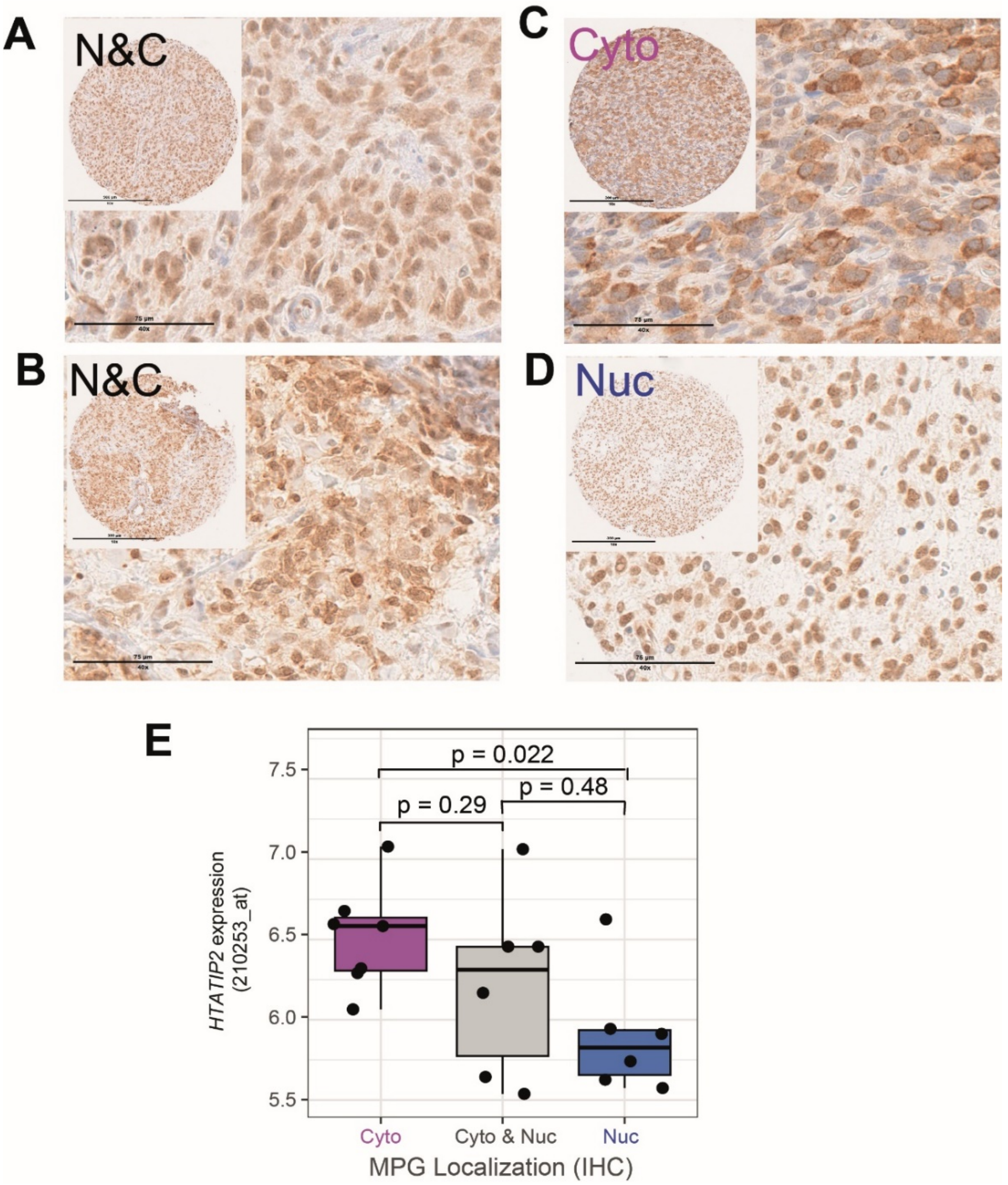

Fig. S1 MPG expression patterns in GBM (related to Fig. 1)

Representative images for distinct patterns of MPG expression in GBM (anti-body against MPG). **A** and **B,** nuclear and cytoplasmic (N&C) MPG expression. Cytoplasmic (Cyto) MPG, **C**; and nuclear (Nuc) MPG expression, **D**, respectively. Size bar; 75 μm / 40x; inserts, 300 μm / 10x **E**, Box plot for *HTATIP2* expression (AFFY 210253_at) stratified by MPG expression patterns. Of note, GBM with both, Nuc & Cyto (grey) MPG expression patterns, exert the full range of *HTATIP2* expression of the combined Nuc (blue) and Cyto (pink) GBM samples, illustrated by boxplot representation where the rectangle, horizontal dark line and vertical dark line correspond to interquartile range (IQR), median and the distance between minimal and maximal values, respectively. The observations (black points) are superimposed on the boxplot representation.

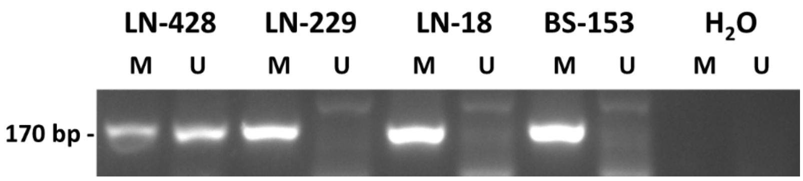

Fig. S2 *HTATIP2* promoter methylation in GBM cell lines.

Methylation Specific PCR (MSP) for *HTATIP2* reveals a signal for methylated and unmethylated (U) *HTATIP2* in LN-428 that expresses *HTATIP2,* while the other three cell lines show exclusively methylated (M) *HTATIP2*, explaining lack of expression.

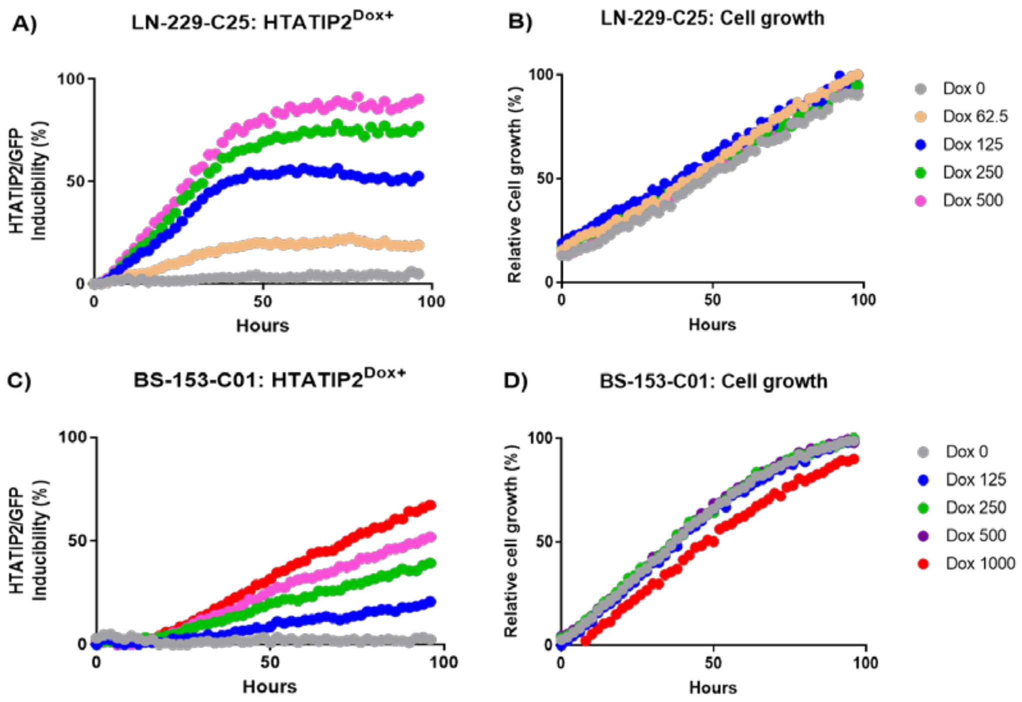

Fig. S3 HTATIP2 is induced by Doxycycline in a dose-dependent manner without affecting cell growth.

A HTATIP2-GFP was induced by Doxycycline (Dox, 0 to 500ng/ml) in a dose-dependent manner as visualized by GFP for (LN-229-C25-HTATIP2^{Dox}) monitored by Incucyte Imaging over a time course of 96 h. **B** Cell growth was not affected by Dox-induced HTATIP2 expression as determined by monitoring cell confluency using phase contrast. **C** and **D**, corresponding experiments for BS-153, clone 01 (BS-153-C01-HTATIP2^{Dox}) (Dox, 0 to 1000 ng/ml), recapitulating the dose-dependent LN-229 clone 25 induction of HTATIP2-GFP in absence of an effect on cell proliferation. Similar results were obtained by other clones in both lines. Of note, the experiments in absence of Dox indicate the tightness of the inducible system.

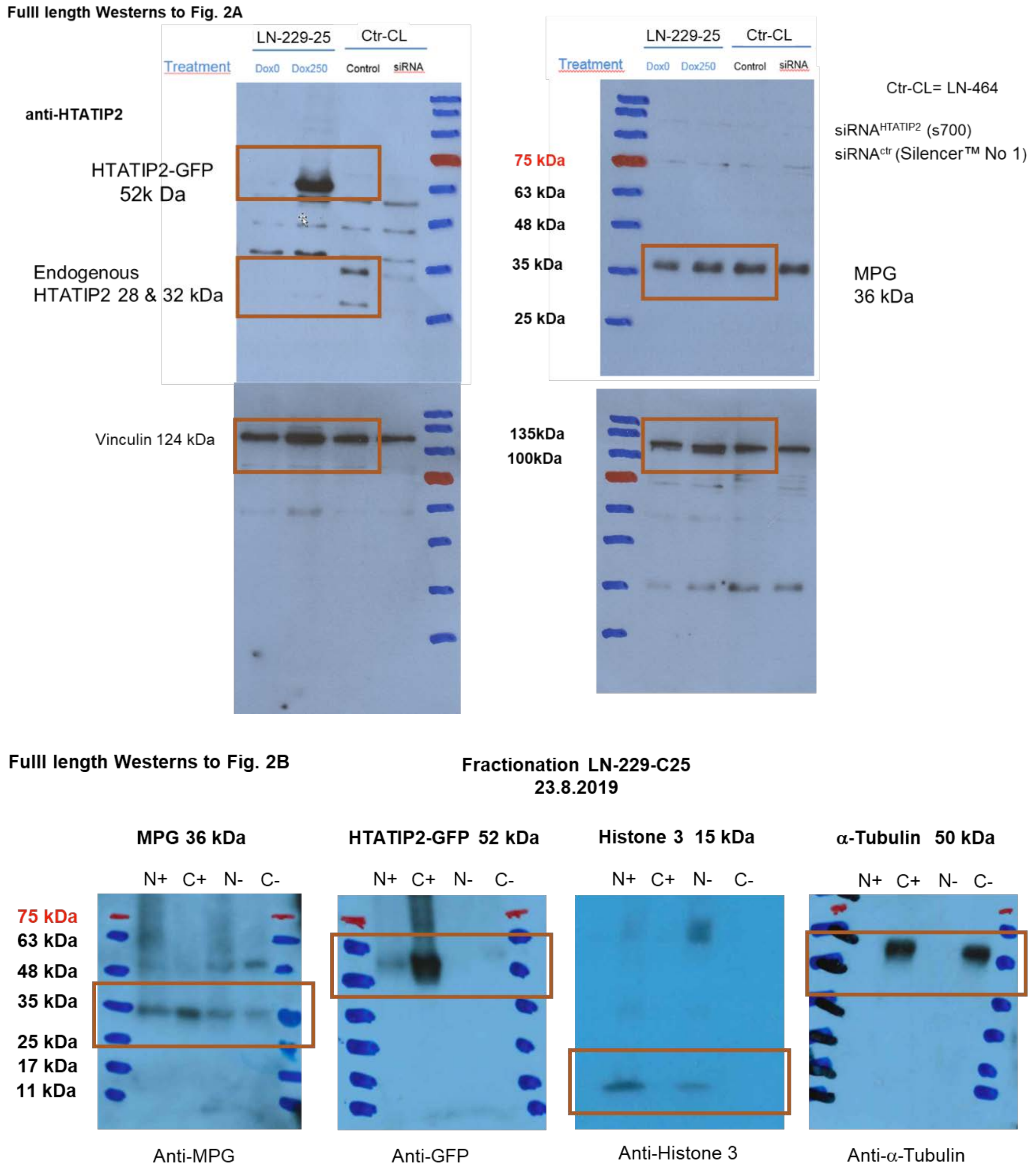

Fig. S4. Full length Westerns corresponding to Fig. 2A and B.

To Fig. 2A: Western blots were probed with Antibodies (Abs) against HTATIP2 (28, 32 kDa), MPG (33kDa), Vinculin (124 kDa). Dox, Doxycycline; LN-229-25, LN-229 clone 25 DOX-inducible *HTATIP2*; CL, cell-line (positive control for endogenous HTATIIP2 expression, LN-464); siRNA^{HTATIP2}, s700; siRNA^{ctr}, Silencer™ No 1.

To Fig. 2B: Western blots were probed with Abs against MPG, Histone 3 (15 kDa), α-Tubulin (50 kDa), GFP (detection of HTATIP2-GFP fusion protein, 52 kDa). N, Nuclear fraction;; N+, Dox 250ng/mL-treated; N-, Untreated; C, Cytoplasmic fraction; C+ (Dox 250ng/mL-treated), C- (Untreated)

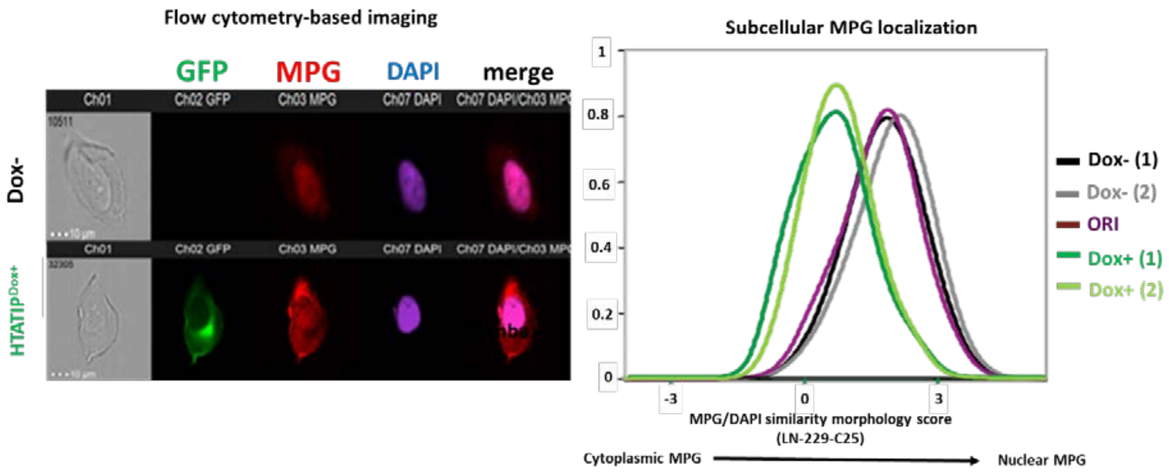

Fig. S5. HTATIP2 induces cytoplasmic retention of MPG.

Single cell imaging and quantification by ImageStreamX MarkII. Representative images of LN-229-C25^{Dox} cells with or without HTATIP2 expression (Dox+, Dox-) visualized for GFP (HTATIP2-GFP fusion protein), immunofluorescence for MPG (red), DAPI (blue), and as merged image. The frequency distribution of the similarity score between MPG and DAPI from every single cell in the whole population: Dox+ (2 populations, dark and light green), Dox- (2 populations, black, grey), and non-transduced mother cell line LN-229 (purple). HTATIP2^{Dox+} expression was associated with a shift of the frequency distribution to the left, towards decreasing similarity score or cytoplasmic MPG. Representation of ImageStream images and quantification by IDEAS 6.2 (experiments in triplicate, total 42547 cells). Scale bar 10 µm.

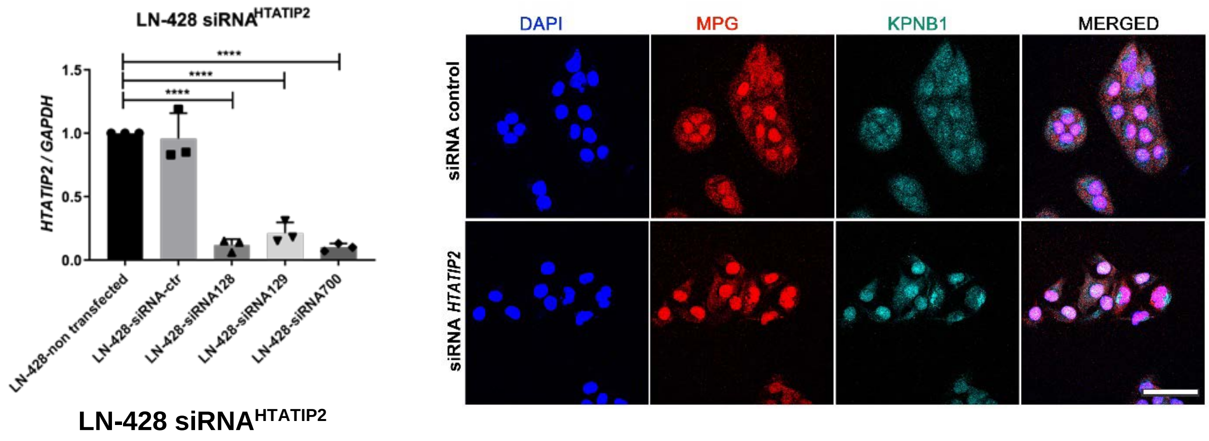

A **B**

Fig. S6. Depletion of *HTATIP2* by siRNA in LN-428

A *HTATIP2* mRNA expression upon knockdown with three anti-*HTATIP2* siRNAs in LN-428 (expresses endogenous *HTATIP2)*. *HTATIP2* mRNA levels were assessed by qRT-PCR 48 h after transfection with 2 nM siRNA (siRNA128, s230128; siRNA129, s230129; siRNA700, s20700; and control siRNA/scrambled (Silencer™ Select Negative Control No. 1) and compared to non-transfected cells. Data are shown as mean±s.d, ****p<0.0001 (One-way ANOVA test). **B** Representative images of LN-428 cells transduced with siRNA s700 or control siRNA (scramble) visualized for DAPI, blue; MPG, red; KPNB1, cyan; or merged. LSM 880 microscopy. Scale bar 20 μm.

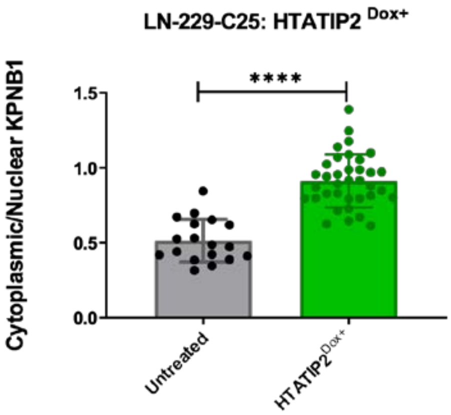

Fig. S7. HTATIP2 inhibits nuclear transport of KPNB1

Quantification of Cytoplasmic /Nuclear KPNB1 in untreated and HTATIP2^{Dox+} induced LN-229-C25 cells. Images were acquired on a LSM 880 microscope. t test ****, p<0.0001.

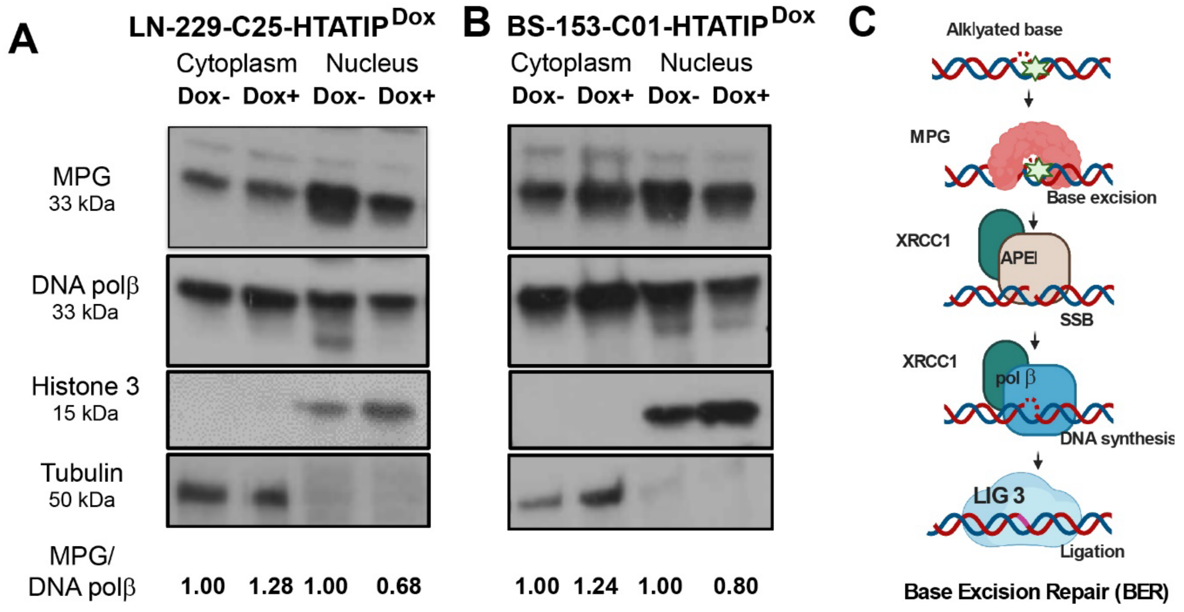

Fig. S8. Effect of HTATIP2 on the nuclear and cytoplasmic ratio of MPG/ DNA Pol β

A LN-229-C25^{HTATIP2Dox} and **B** BS-153-C01^{HTATIP2Dox} cells were treated with Dox (Dox+) and fractionated into their nuclear (N) and cytoplasmic fraction (C) as illustrated by Western blot. The nuclear fraction was normalized using Histone 3 (15 kDa) and the cytoplasmic fraction with Vinculin (124 kDa). The ratio of MPG / DNA Pol β was calculated for the nuclear and the cytoplasmic fraction, based on densitometry, in function of HTATIP2 induction (Dox+/-), normalized to the respective fraction of untreated cells (Dox-). HTATIP2 expression (Dox+) reduced the relative MPG / DNA Pol β ratio in the nucleus, while enhancing it in the cytoplasm. **C** Schematic representation of the Base Excision Repair (BER) pathway to illustrate the role of DNA polymerase β (DNA Pol β). The DNA N-glycosylase MPG recognizes bases alkylated at N-positions (N7-meG, N3-meG, and N3-meA) and hydrolyses the deoxyribose N-glycosidic bond, leaving behind apurinic and apyrimidinic (AP) sites that are toxic intermediate lesions of BER. The generated AP sites will then be cleaved by the endonuclease activity of APE1, leaving a gap in the single strand DNA. This gap will be filled up by DNA Pol β which is a rate-limiting step [3]. Illustration in **C** was created with BioRender.com (Agreement number *GG25KERU3O)*.

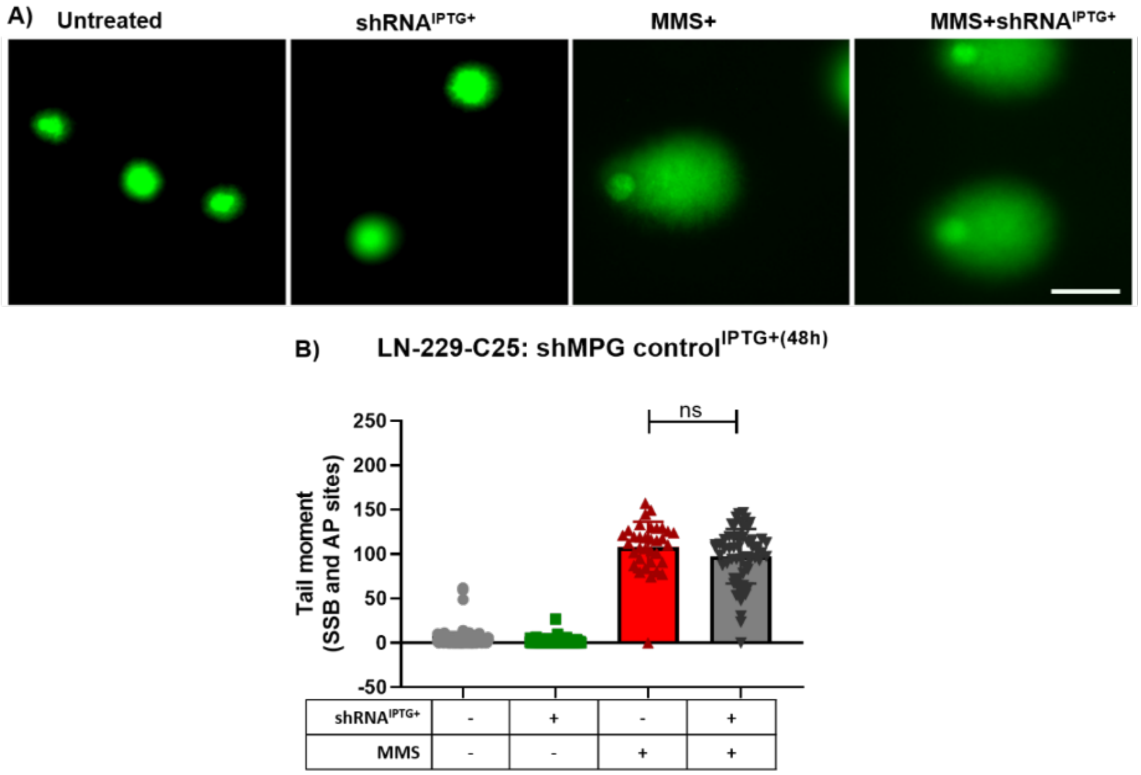

Fig. S9. Control anti-*MPG* shRNA did not affect MMS response in LN-229-C25.

Comet assay quantifies comet tail moment, which reflects DNA single strand breaks and Abasic or Apurinic/apyrimidinic (AP) sites under four treatment conditions: Untreated (grey), shRNA control^{IPTG+} (green), MMS 200nM (red) and combination (black). **A** Representative images of LN-229-C25 after 48h treatment with or without MMS are shown. **B** Quantification of the comet tail moment. This is the shRNA control for the experiment shown in Fig. 4B and C. One experiment.

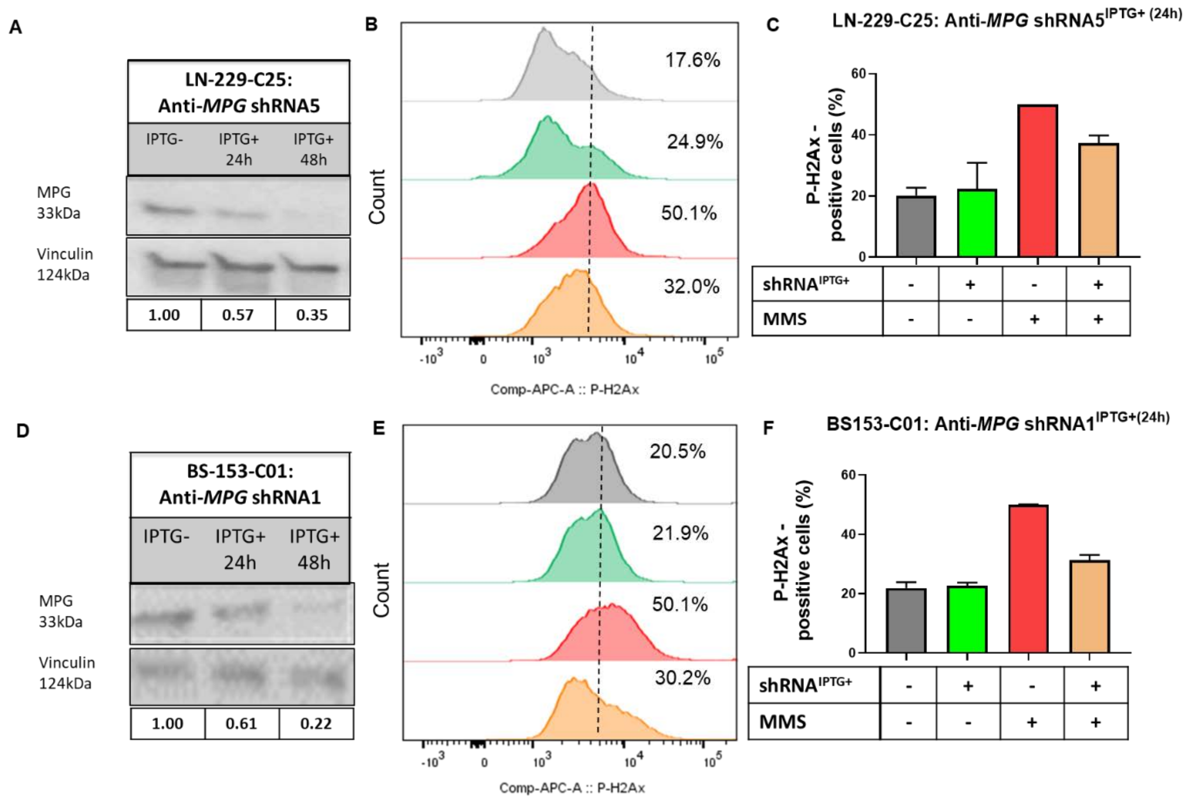

Fig. S10. Depletion of MPG reduces effect of MMS on DNA DSB.

Induction of anti-MPG shRNA^{IPTG} with IPTG [500ng/ml] depleted cells of MPG with an efficiency of 65% in LN-229-C25-shMPG5 and 78% in BS-153-C01-shMPG1 after 48 h, respectively, as indicated by Western **(A** & **D)**. Cells were depleted or not for MPG and treated with MMS for 24 h and P-H2Ax was measured by FACS. The frequency distributions of the P-H2Ax signal, representing DNA DSB are visualized (**B** and **E)**. A threshold for DNA DSB damage was defined at 50% P-H2Ax positive cells in the MMS+ treatment group. Depletion of MPG resulted in an attenuation of DNA DSB induced by MMS in LN-229-C25-shMPG5^{IPTG+} [200nM] and BS-153-C01-shMPG1^{IPTG+} [100 nM] **(C** and **F).** Quantification of DNA DSB of two biological replicates in two cell lines, respectively, are shown, error bars are SD.

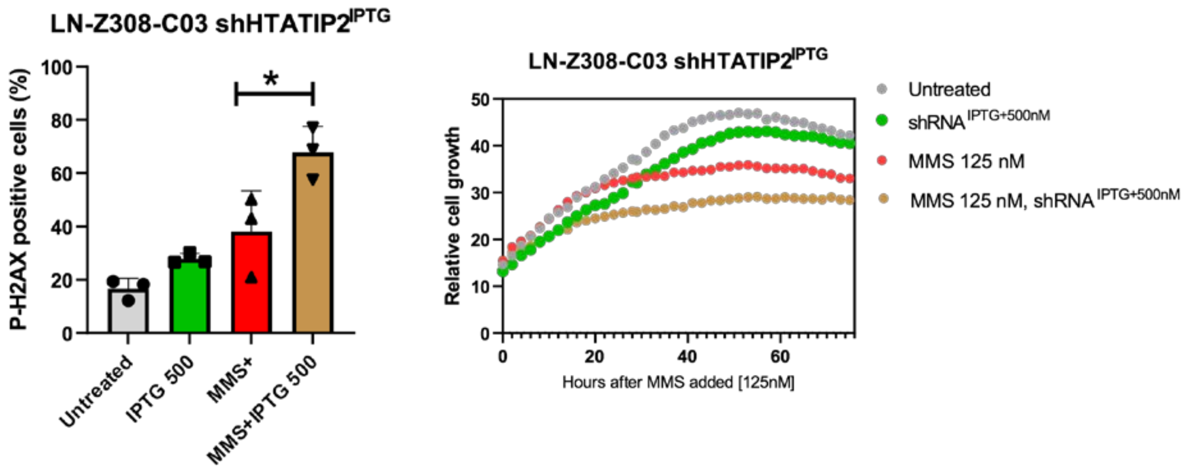

A **B**

Fig. S11. HTATIP2 depletion enhances the MMS induced DNA damage in LN-Z308 cells. The GBM cell line LN-Z308 transduced with the inducible sh1-HTATIP2^{IPTG} was pretreated for 48h with IPTG [500nM] to deplete endogenous *HTATIP2,* followed by treatment with MMS [125nM]. **A** The cells were analyzed by FACS for P-H2AX after 24h treatment with MMS. The mean of 3 technical replicates is shown. Error bars are SD, P-value *, <0.05, unpaired t-test. **B** Relative cell growth was monitored for four treatment conditions over 72h by Incucyte imaging. A representative experiment is shown. Depletion with shRNA2 and 3 were also performed, shRNA1 resulted in most efficient depletion.

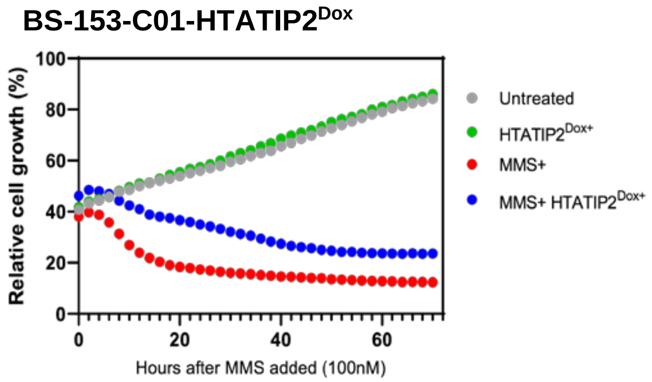

Fig. S12. HTATIP2 attenuates MMS induced reduction of cell growth in BS-153.

BS-153-C01-HTATIP2^{Dox} cells were treated with MMS [100 nM] and monitored for cell proliferation over 72 h by Incucyte imaging, quantified by cell confluency (phase contrast). Cells were induced, or not to express HTATIP2 (Dox+ [500 nM]).

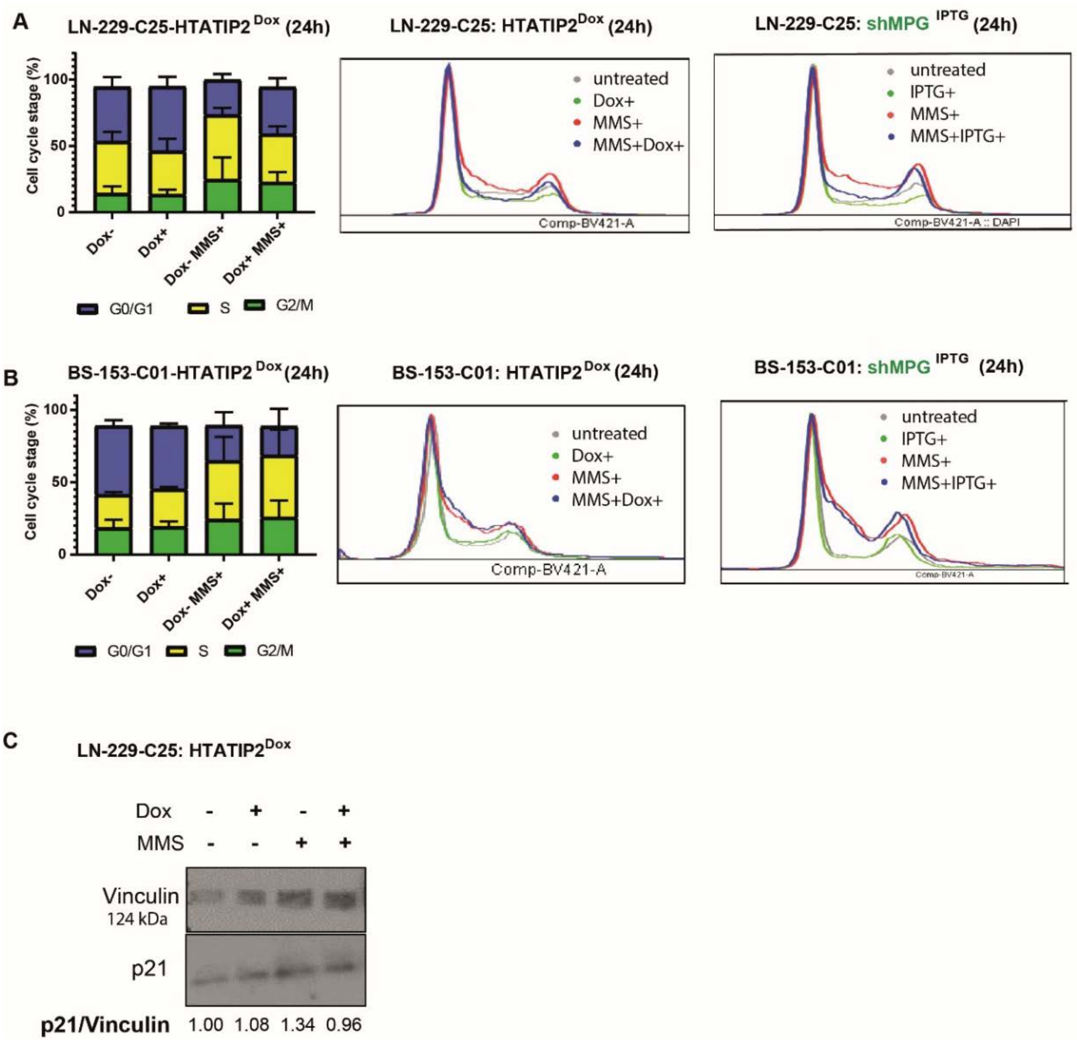

Fig. S13: Cell cycle progression of MMS treated cells in function of HTATIP2.

Cell cycle profile analysis using DAPI for nuclear stain in the HTATIP2-Dox inducible cell lines. **A** LN-229-C25-HTATIP2^{Dox} was treated with MMS [200 nM] for 24 h. Bar charts summarize the cell cycle profiles under 4 treatment conditions computed from 3 biological replicates; G0/G1, green; S-phase, yellow; G2/M blue. A trend for attenuation of the prolonged S-phase was observed in presence of HTATIP2 (Dox+ [250 μg/ml]) expression (p=0.051, multiple t-tests). Overlapping cell cycle profiles from a representative experiment are shown. Untreated, grey; Dox+, green; MMS, red; and combination of MMS+ and Dox+ (blue). **B**, A similar experiment was performed with BS-153-C01-HTATIP2^{Dox} (Dox+, [500 μg/ml]; MMS [100 nM]). The bar chart summarizes the results of 3 biological replicates. The overlapping cell cycle profiles are shown for one representative experiment. **C**, A western blot for LN-229-C25-HTATIP2^{Dox} shows induction of p21 by MMS at 24 h using the 4 treatment conditions as in **A**. HTATIP2 expression attenuates MMS-treatment mediated induction of p21. Relative concentrations are normalized to Vinculin.

Legends of Supplementary Videos

Video S1. Nuclear localization of MPG in absence of HTATIP2 expression in LN-229-C25 (Dox-) cells (3D).

The subcellular localization of MPG in function of HTATIP2-expression is visualized in an animated 3D projection reconstructed from confocal microscopy. In absence of HTATIP2 expression in LN-229-C25 (Dox-) cells MPG showed nuclear subcellular localization. (Supplementary Animation S1 and S2, 3D animations, code; DAPI, blue; MPG, red; HTATIP2-GFP, green)

Video S2. Retention of MPG in the cytoplasm upon expression of HTATIP2 in LN229-C25 (Dox+) cells (3D).

Dox-induced (250 nM) HTATIP2 expression in LN-229-C25 (Dox+) cells led to retention of MPG in the cytoplasm (Supplementary Animation S2, 3D animations, code; DAPI, blue; MPG, red; HTATIP2-GFP, green).

References

1. Lu B, Ma Y, Wu G, Tong X, Guo H, Liang A, Cong W, Liu C, Wang H, Wu M, Zhao J & Guo Y (2008) Methylation of Tip30 promoter is associated with poor prognosis in human hepatocellular carcinoma. Clin Cancer Res 14, 7405-7412, doi: 10.1158/1078-0432.CCR-08-0409.

2. Agnihotri S, Burrell K, Buczkowicz P, Remke M, Golbourn B, Chornenkyy Y, Gajadhar A, Fernandez NA, Clarke ID, Barszczyk MS, Pajovic S, Ternamian C, Head R, Sabha N, Sobol RW, Taylor MD, Rutka JT, Jones C, Dirks PB, Zadeh G & Hawkins C (2014) ATM regulates 3-methylpurine-DNA glycosylase and promotes therapeutic resistance to alkylating agents. Cancer Discov 4, 1198-1213, doi: 10.1158/2159-8290.CD-14-0157.

3. Krokan HE & Bjoras M (2013) Base excision repair. Cold Spring Harbor perspectives in biology 5, a012583, doi: 10.1101/cshperspect.a012583.
